# Nonprostatic diseases on PSMA PET imaging: a spectrum of benign and malignant findings

**DOI:** 10.1186/s40644-020-00300-7

**Published:** 2020-03-14

**Authors:** Felipe de Galiza Barbosa, Marcelo Araujo Queiroz, Rafael Fernandes Nunes, Larissa Bastos Costa, Elaine Caroline Zaniboni, José Flavio Gomes Marin, Giovanni Guido Cerri, Carlos Alberto Buchpiguel

**Affiliations:** Department of Radiology, Hospital Sirio-Libanes, Rua Dona Adma Jafet, 91, Sao Paulo, ZIP: 01308-050 Brazil

**Keywords:** Prostate cancer, Positron emission tomography, (68)Ga-PSMA

## Abstract

PSMA PET imaging was originally used to assess biochemical recurrence of prostate cancer (PCa), but its clinical use was promptly extended to detection, staging and therapy response assessment. The expanding use of PSMA PET worldwide has also revealed PSMA ligand uptake in diverse nonprostatic diseases, which raised questions about the specificity of this imaging modality. Although not very common initially, a growing number of pathologies presenting PSMA uptake on PET have been reported in the last few years, and a proper interpretation of PSMA PET imaging findings suddenly became challenging and, to some extent, confusing. Compared to cytoplasmic PSMA expression in nonprostatic cells, the molecular features of apical PSMA expression in PCa cells can help to distinguish these various conditions. Correlations of imaging findings to patient history, to the expected pattern of disease spread and mainly to computed tomography (CT) and/or magnetic resonance imaging (MRI) characteristics will reinforce the distinction of lesions that are more likely related to PCa from those that could lead to an incorrect diagnosis. The overall benefits of endothelial PSMA expression, which is associated with the neovasculature of malignant neoplasms, will be highlighted, stating the potential use of PSMA ligand uptake as a theranostic tool. This review aims to cover the collection of nonprostatic diseases, including benign and malignant tumors, in a didactic approach according to disease etiology, with discussion of bone-related conditions and inflammatory and infectious processes.

## Background

**Fig. 1:**
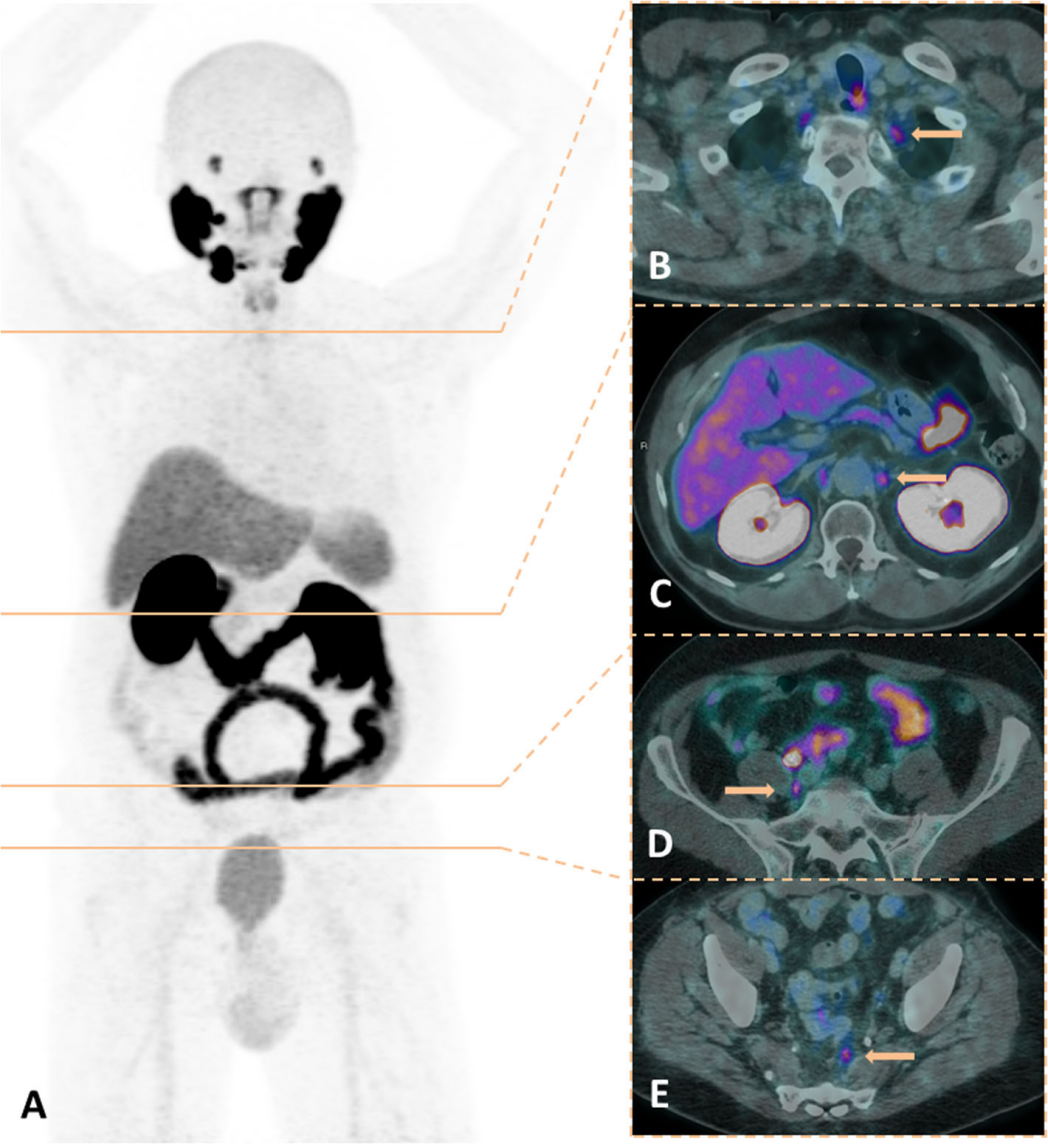
An overview of physiological PSMA uptake in sympathetic ganglia. ^68^Ga-PSMA-PET MIP (**a**) with corresponding levels on fused PET/CT images (**b**, stelate ganglia; **c**, celiac ganglia; **d**, hypogastric ganglia; **e**, sacral ganglia)

**Fig. 2:**
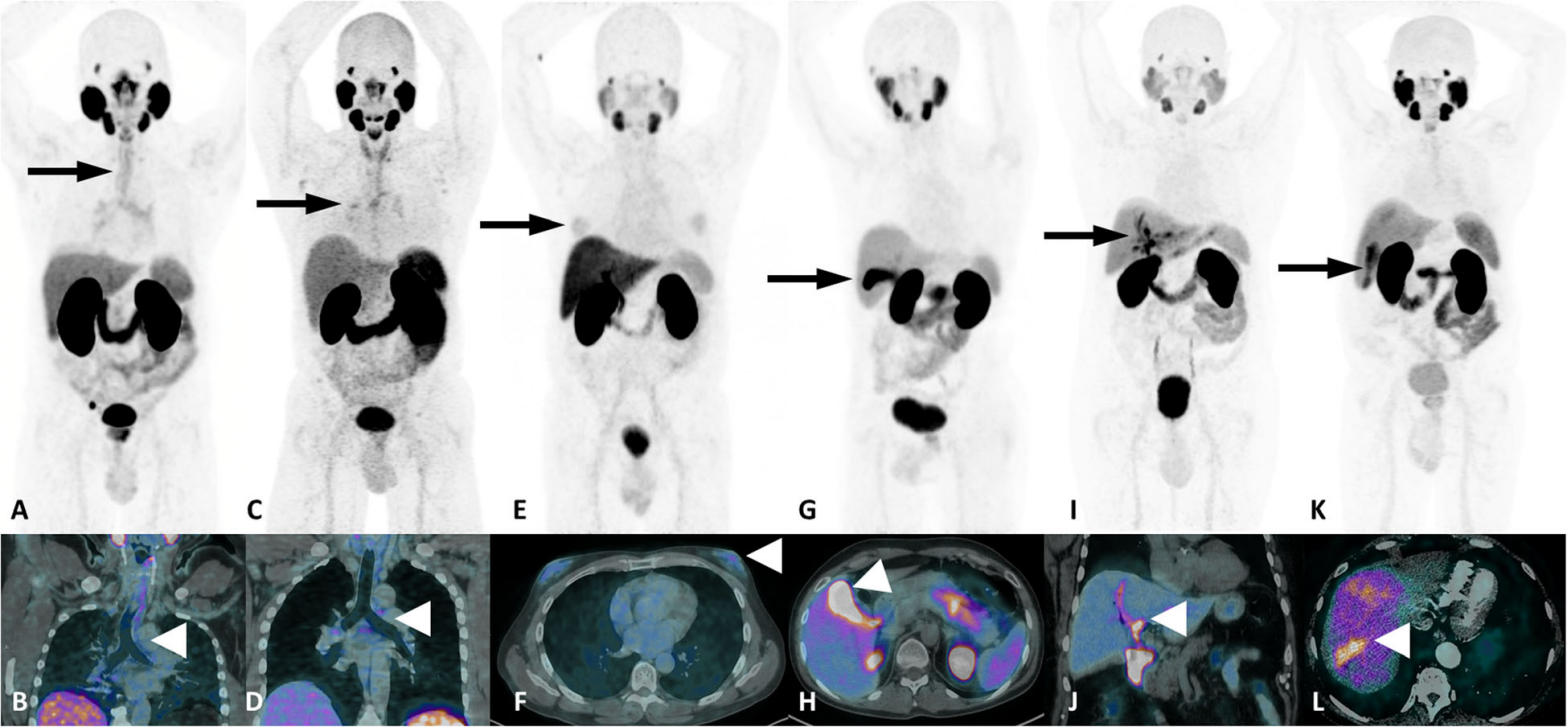
A miscelanea of normal variants of PSMA uptake. ^68^Ga-PSMA-PET MIP images on top (**a**, **c**, **e**, **g**, **i** and **k**) and corresponding fused PET/CT images on bottom (**b**, **d**, **f**, **h** and **l**) showing trachea and bronchi (**a**, **b**), mediastinal lymph nodes (**c**, **d**), gynecomastia (**e**, **f**), gallbladder (**g**, **h**), billiary tract **(i**, **j**) and liver perfusion defect (**k**, **l**)

Prostate cancer (PCa) management has been revolutionized with the advent of prostate-specific membrane antigen (PSMA) positron emission tomography (PET) imaging. Accordingly, the literature has been flooded with an enormous diversity of cases of incidentally detected PSMA uptake in nonprostatic conditions, which led to questioning the specificity of PSMA PET results [1, 2]. However, a recent meta-analysis including 4790 patients who underwent ^68^Ga-PSMA PET/CT for different indications showed a per-lesion specificity of 99% [3]. Therefore, the nonprostatic diseases that exhibit PSMA uptake on PET imaging represent an uncommon finding that need to be recognized not only to avoid misdiagnosis but also to allow for correct therapy planning. On the other hand, although nonprostatic PSMA uptake might hamper the diagnostic performance of PSMA PET imaging, the receptor expression observed in some malignant tumor cells broadens the application of its use in diagnosis or even therapeutic approaches using PSMA-targeted radionuclide therapy [4]. Since it is a new imaging modality, even the normal biodistribution of PSMA on PET imaging is often revisited, and other anatomical sites are related as normal variants. Intense PSMA uptake is usually observed in lacrimal, parotid and submandibular glands, and in the small intestine, kidneys, liver, spleen and bladder, while mild to moderate uptake may be observed in nasal and esophageal mucosa; the vocal cords, gallbladder and biliary tract; tracheal and proximal bronchi; mediastinal, axillary and inguinal lymph nodes; gynecomastia; and sympathetic ganglia, such as stellate, celiac, hypogastric and presacral [5–7]. An overview of these conditions with physiological and benign PSMA uptake is shown on Figs. 1 and 2.

In this review, the nonprostatic conditions exhibiting PSMA uptake were divided in a didactic manner according to the underlying etiologies of the diseases: bone-related conditions, inflammatory and infectious processes (including surgery-related findings) and benign and malignant tumors. It is important to note that all of our patients underwent PET imaging with ^68^Ga-PSMA-11 (herein referred to as only PSMA) as the PET tracer, but the rationale of nonprostatic uptake is likely transferable to all PSMA-based available PET tracers (e.g., ^68^Ga-PSMA-I&T, ^68^Ga-THP-PSMA ^18^F-DCFPyL, and ^18^F-PSMA-1007), based on the biological mechanism of PSMA uptake hereinafter detailed. The key features for differential diagnosis of metastatic diseases will also be thoroughly highlighted to warrant an appropriate imaging interpretation.

## Main text

### Mechanism of PSMA uptake

PSMA is a type II (i.e., integral) transmembrane glycoprotein that was discovered in 1987 in metastatic PCa cell lines [8]. PSMA is encoded by the FOLH1 gene in the short arm of chromosome 11 and is formed by 750 amino acids (aa), divided into intracellular, transmembrane and extracellular regions. The last region is the largest, with 707 aa, and contains specific enzymatic domains, which are the main target for current PSMA-ligand imaging and therapy [9]. In contrast with other clinically relevant prostate-related antigens, such as prostate-specific antigen or prostatic acid phosphatase, which are secretory proteins, the PSMA transmembrane conformational structure enables it to exhibit an internalization functionality by means of endosomal complexes, which is a highly attractive feature for targeted diagnostic and therapeutic approaches, especially with radiopharmaceuticals [10, 11]. PSMA functions are not completely understood, but reported functions include enzymatic peptidase activity related to folate and glutamate metabolism, as well as activation of signaling pathways (e.g., Akt and MAPK) involved in cell proliferation and survival [12]. PSMA expression occurs in normal epithelial prostate cells and is highly upregulated in PCa cells according to the biological aggressiveness of the disease, generally becoming more intense in high Gleason score, poorly differentiated, castration-resistant and metastatic tumors. Despite this well-known association, the exact role of PSMA in PCa cell differentiation *status*, proliferation and metastatic potential remains unclear [13].

However, in contrast to the idea induced by its name, PSMA expression is not exclusive to prostate cells and can be found in several other tissues and/or conditions, including normal nonprostatic epithelial cells, inflammation/infection, nonprostatic neoplastic cells and nonprostatic tumor-associated neovasculature. To better understand this variability in PSMA expression and its potential consequences on PSMA-based image interpretation, the following concepts should be kept in mind by readers.

#### Apical membrane vs cytoplasmic PSMA expression

**Fig. 3:**
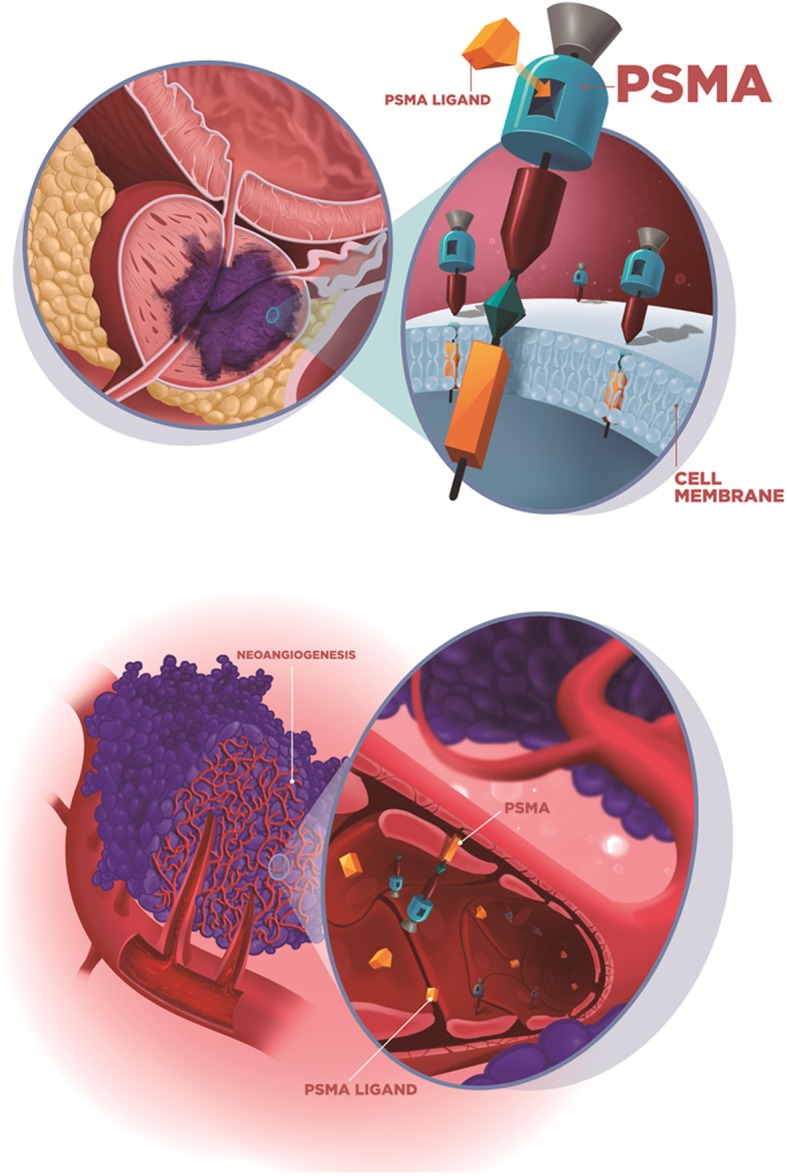
Ilustration of the two main mechanism os PSMA expression that explains the radiotracer uptake in prostate cancer (**a**) and other non-prostatic causes with endothelial expression (**b**)

From the structural perspective of an epithelial cell, PSMA expression may occur in the apical (i.e., luminal) membrane, in the cytoplasm or in both. Apical membrane expression is the prototype of PSMA expression, occurring in normal prostatic epithelial cells, and mostly in PCa cells (Fig. 3a)**.** Apical expression is markedly amplified in PCa cells in comparison with normal prostatic epithelial cells and other tissues/neoplastic cells. This type of expression leads to intense PSMA accumulation on both immunohistochemistry and imaging studies. A study of PSMA expression with immunohistochemistry in 3161 varied tumors found that apical membrane expression tends to be more intense and occurs only in PCa because all of the nonprostatic tumors exhibited PSMA expression in the cytoplasm, which is normally less intense than that in the membrane [14]. Interestingly, Osman and colleagues, who studied synchronic malignancies that were detected in a cohort of 764 patients that underwent PSMA PET, reached similar findings some years later. A higher uptake (despite some overlap, especially in lung lesions) in PCa lesions confirms higher specificity of PSMA overexpression to detect PCa lesions, which was likely an observation of the same phenomena in the light of a new technology (7). More recently, Rischpler et al recalled this theme when studying PSMA ligand uptake patterns in sympathetic ganglia and showed consistently lower PSMA ligand uptake in these structures than in with PCa metastatic lesions [15]. Despite the unquestionable consistency of this finding across several studies, no validated threshold of PSMA ligand uptake was found until now to safely differentiate PCa from nonprostatic lesions, highlighting the importance of recognizing morphological and biological patterns of different diseases to produce clinically valuable reports.

#### Endothelial expression

Endothelial PSMA expression has been extensively studied and occurs in almost all nonprostatic solid tumors associated with neovasculature but not in benign endothelial tissue, raising some hypotheses about the role of PSMA in neoangiogenesis and vascular growth factors regulation [12, 16] (Fig. 3b). Therefore, PSMA ligand uptake by nonprostatic solid tumors (benign and malignant) in PET imaging must be interpreted with this knowledge to help readers pay attention to histological, imaging and clinical information about tumor neovascularization. Important remarks are the lack of PSMA expression in the endothelial cells of normal tissues and, interestingly, in PCa-associated neovasculature.

#### Inflammation/infection expression

Few data are available about the mechanism of PSMA expression by immune cells. It is possible that neovascularization also plays a role in this scenario as well as an increased availability of PSMA ligand to the site of inflammation/infection due to an increase in regional blood flow/vascular permeability. Additionally, some inference about macrophage folate receptors and their potential implications in PSMA ligand imaging has been suggested [17]. However, in the PSMA ligand PET era, an increasing number of reports of false-positive findings in inflammatory/infectious diseases have been published, making this a practical question that requires the attention of readers in daily reporting.

### Nonprostatic diseases

Lesion evaluation can be challenging in specific situations, and practitioners should use a combination of aspects: a) molecular (i.e., are the intensity, extension, and semiquantitative patterns different from those of other lesions or from those expected to occur in PCa?); b) morphological (i.e., are the CT/MRI findings typical of a specific neoplasm or of a PCa lesion?); c) biological (i.e., does this lesion reasonably fit with known PCa staging, predicted spread, and/or PSA values?); and d) clinical (i.e., is there some underlying condition/comorbidity that can be linked to this lesion?). Of course, there is no absolute discriminative answer generated by these questions, but available data about patterns of uptake (tends to be much higher in PCa, despite some potential overlap in organs such as the lungs or liver), knowledge of CT/MRI morphological disease patterns and the understanding of the natural history of various neoplasms allow interpreters to produce more accurate predictions, for example, indicating biopsies with more effectiveness. A list including all the reported cases of nonprostatic diseases with PSMA ligand uptake on PET imaging is summarized in Table 1 [4, 17–32].

**Table 1:** A list of published case reports of nonprostatic diseases presenting PSMA uptke according to the etiology

	Bone-related conditions	Inflammatory and infectious processes	Benign neoplasms	Malignant neoplasms
Central and peripheral nervous system	–	Neurocysticercosis	Meningioma Schwannoma Peripheral nerve sheath tumor Neurofibromas	Gliomas
Cervical	–	–	Thyroid and parathyroid adenomas	Head and neck Squamous cell carcinoma Adenoid cystic carcinoma Thyroid cancer
Thoracic	–	Opacities and bronchietasis Sarcoidosis Tuberculosis Anthracosilicosis Berylliosis	Elastofibroma dorsi Pseudoangiomatous stromal hyperplasia of the breast Thymoma	Breast cancer Lung cancer Thymoma Mesothelioma
Abdominal	–	Postsurgical inflammation Liver and splenic sarcoidosis Diverticulosis Amyloidosis of seminal vesicles Anal fistula	Angiolipoma Hepatic and splenic hemangiomas Adrenal adenoma Pancreatic serous cystadenoma	Hepatocellular carcinoma Cholangiocarcinoma Adrenocortical carcinoma Renal cell carcinoma Transitional cell carcinoma Pancreatic carcinoma Neuroendocrine tumor Gastric GIST Gastric adenocarcinoma Bladder Paraganglioma Cervical carcinoma Ovary cancer Endometrium cancer Vulvar carcinoma
Skeletal, soft-tissues and vascular	Osteomyelitis Fracture Paget’s Disease Hemangioma Osteogenerative Fibrous Osseous Defect Fibrous Dysplasia Sacral insufficiency after RT Osteochondroma Multiple Myeloma Polycythemia Rubra Vera Myelodysplasia	Nodular fasciitis	Dermatofibroma Acrochordon Fibromatosis Desmoid tumor Intramuscular myxoma Hemangiopericytoma Nasal Angiofibroma Angiolipoma Acrochordon Fibromatosis	Lymphoma Osteosarcoma Ewing sarcoma Melanoma Malignant nerve sheath tumor Multiple Myeloma Other soft tissue sarcomas

#### Bone-related conditions

Bone is the most common site of metastatic hematogenous spread in PCa patients. PSMA PET is considered the most accurate and sensitive method not only to evaluate skeletal involvement [33], but also to possibly differentiate ambiguous finding and diagnose benign pathologies.

There are no specific features of PCa bone metastases, but it customarily appears in a high-risk PCa patient presenting multiple blastic lesions predominantly in the pelvis and spine due to the particularities of bone spreading [34]. PSMA avidity has a tendency of being higher in regard to appraising malignant bone lesions in contrast to benign pathologies, which usually present low to moderate uptake. Castrate-resistant prostate cancer patients tend to have metastases with higher PSMA expression compared to those with hormone-sensitive cancer. Therefore, in this specific clinical setup, it is even more likely to expect high PSMA avidity on PCa bone lesions.

The morphological aspects on CT, such as the number of lesions, type of margin, pattern of bone destruction, type of periosteal reaction and presence of an associated soft tissue mass, should always be taken into consideration when evaluating a bone lesion. A benign appearance customarily presents as a slow-growing lesion with geographic destruction, sharply demarcated margins and smooth and uninterrupted periosteal reaction without soft tissue mass.

Osteodegenerative changes are very common among PCa patients, but only very few of them may present mild PSMA uptake, especially in the spine. The clue for diagnosis is the typical morphological appearance of joint space narrowing with sclerosis and osteophytosis.

**Fig. 4:**
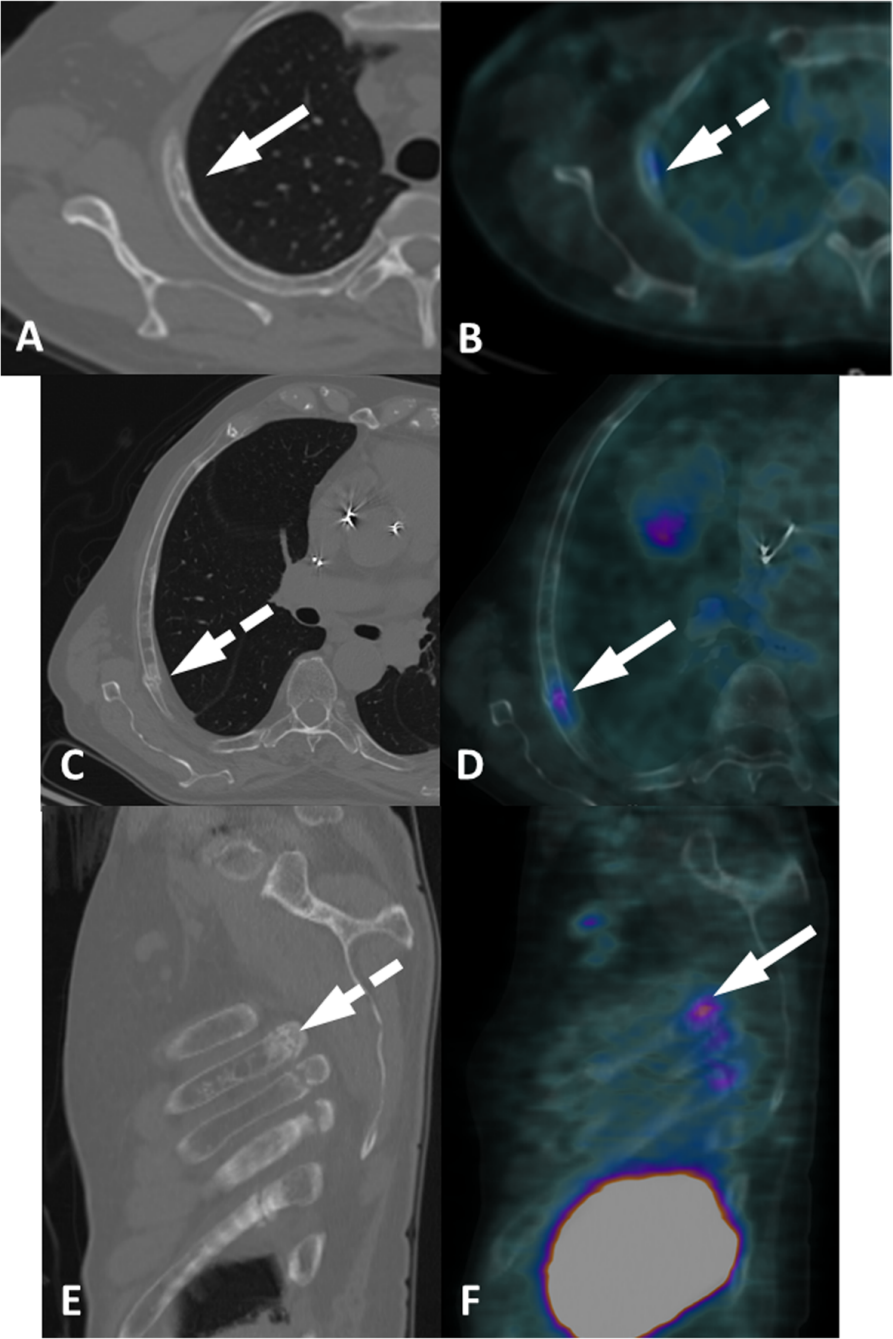
Focal PSMA uptake in the ribs in two different patients. Patient 1, fibrous osseus defect (**a**, **b**). Axial CT (**a**) and axial ^68^Ga-PSMA-PET/CT (**b**) show a single focus of faint uptake (dashed arrow) in a typical fibrous osseous defect of the third right rib (arrow). Patient 2, ribs fractures (**c-f**). Axial CT (**c**), sagittal CT (**e**), axial ^68^Ga-PSMA-PET/CT (**d**) and sagittal ^68^Ga-PSMA-PET/CT (**f**) show mild uptake (arrows) in continuous ribs fractures (dashed arrows)

Another very common finding on PSMA PET imaging is a single mild tracer uptake in the ribs (Fig. 4). It is usually not related to PCa and does not require further investigation (i.e. bone scan or MRI) as the dedicated CT from PET/CT is often sufficient for a confident diagnosis. In few unclear cases a dedicated thoracic CT with a bone filter should offer a better morphological correlation, such as small fibrous osseous defect and a tiny fracture. It is also important to apply a close correlation to the patient’s clinical history, for instance, in cases of recent trauma in the thorax.

**Fig. 5:**
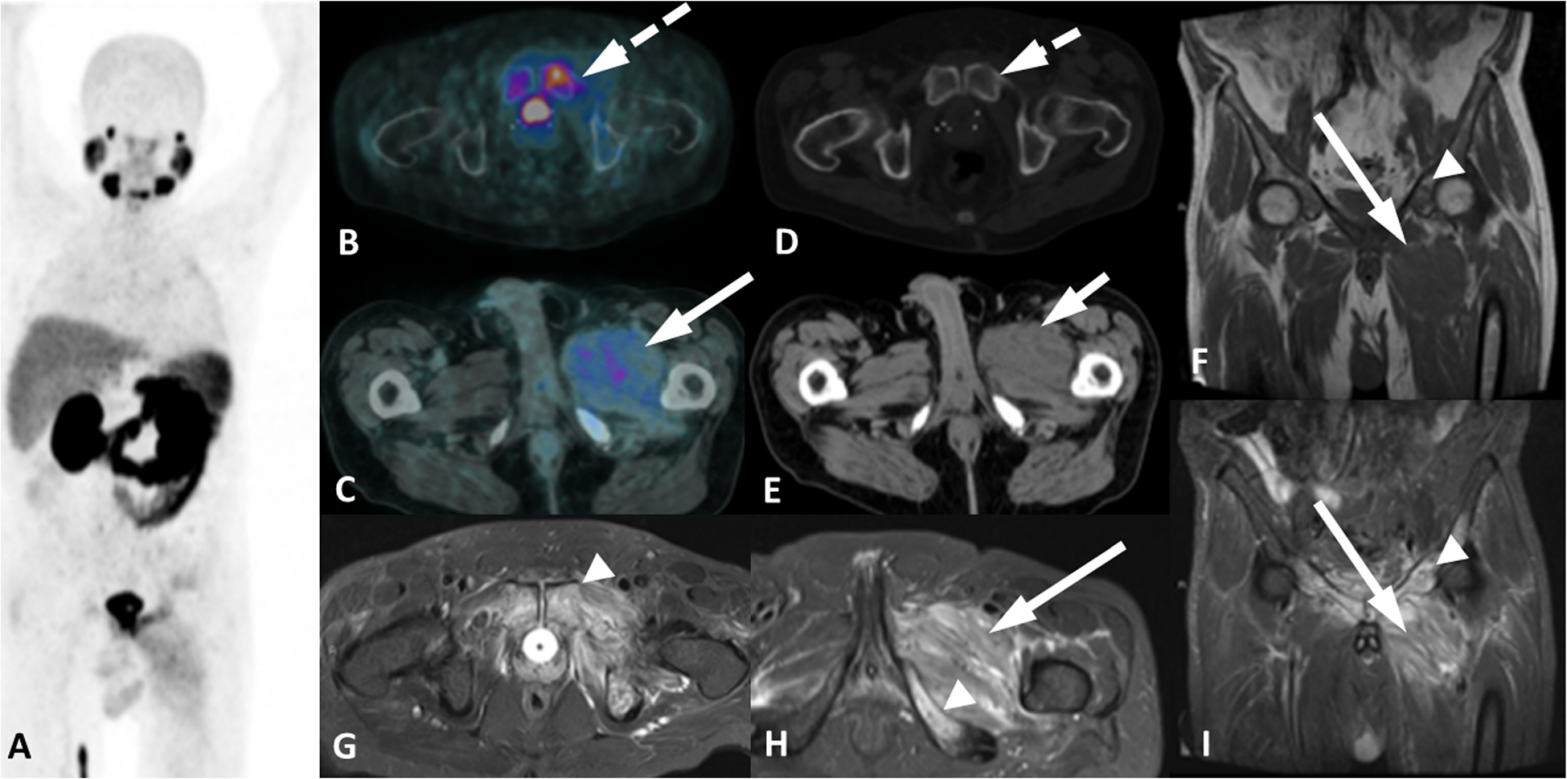
Osteomyelitis with PSMA uptake. ^68^Ga-PSMA-PET/CT MIP (**a**), axial PET/CT (**b**, **c**) and axial CT (**d**, **e**) images show a lytic lesion in the left pubic bone with bilateral diffuse bone sclerosis (small dashed arrow) with mild uptake (dashed arrow). Additionally, there is edema in the right adductors muscles (arrow) with discrete and heterogeneous uptake (short arrow). These findings - osteomyelitis (arrowheads) and inflamation in the adjacent soft-tissue (long arrows) - were confirmed on MR images in coronal T1w (**f**) axial (**g**, **h**) and coronal (**i**) T2w Fat Sat

PSMA uptake in osteomyelitis might be related to transient vascular distress in a patient with a typical clinical history (systemic inflammation, past trauma or ulcer). In these cases, it is mandatory to correlate PET findings with CT (inhomogeneous osteosclerosis with bony sequestrum) and/or MRI (low T1 signal within the bone), indicating cortical destruction and bone marrow edema. Abscess and soft tissue collections may be observed after injection of gadolinium (Fig. 5).

**Fig. 6:**
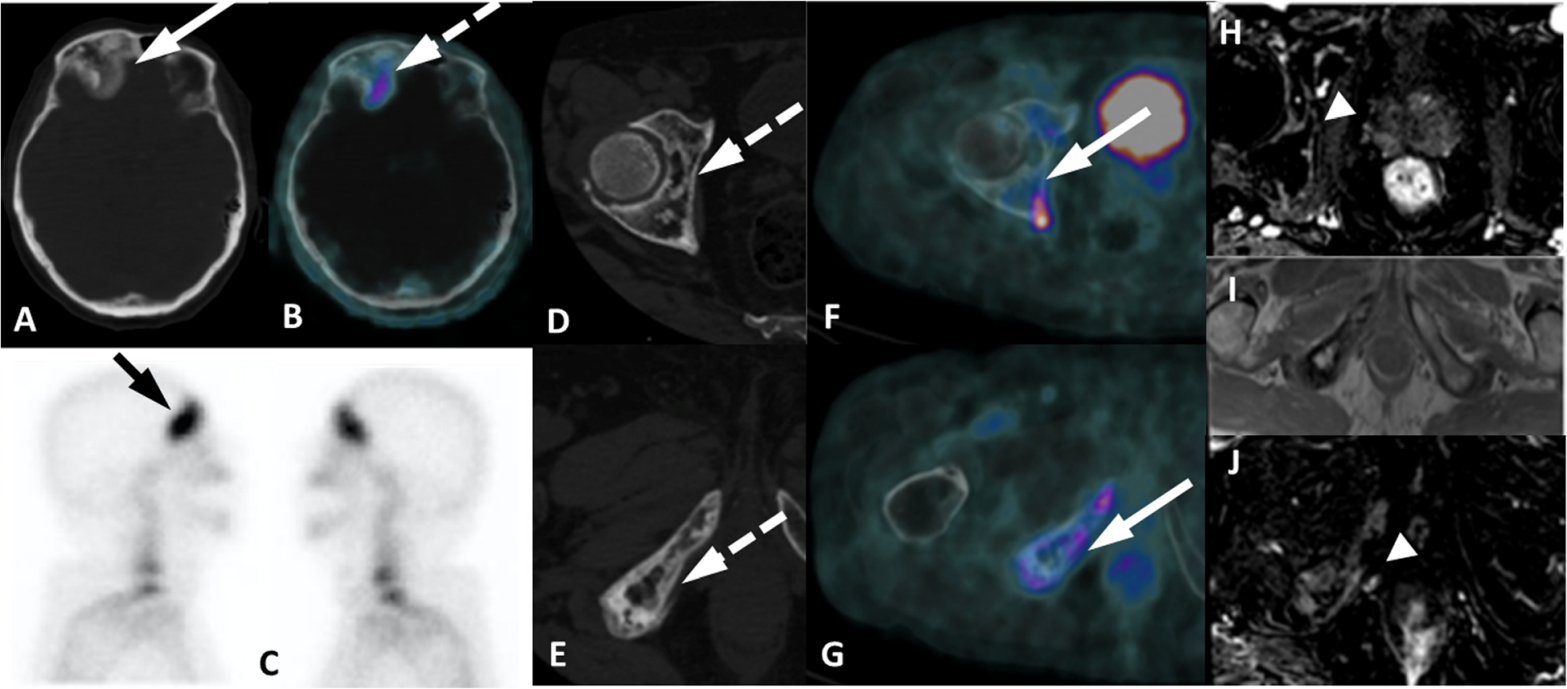
Bone PSMA uptake in correlation with morphology and other imaging modalities in two different patients. Patient 1, fibrous dysplasia (**a-c**). Axial CT (**a**) and axial ^68^Ga-PSMA-PET/CT (**b**) show a homogeneously sclerotic lesion with well-defined borders (arrow) and mild PSMA uptake (dashed arrow), suggestive of fibrous dysplasia. Previous MDP-bone scan lateral images (**c**) showed an intense osteogenic reaction (black arrow) in the right frontal bone, which is impossible to differentiate from a bone metastatis without a CT. Patient 2, Paget’s disease (**d-j**). Axial CT (**d, e**), axial ^68^Ga-PSMA-PET/CT (**f, g**) and axial T1w MR (**i**) images show mild uptake (arrows) in the cortical thickening and sclerosis of the right ischium (dashed arrows). Axial perfusion (**h, j**) MR images show an increased vascularization exactly in the same areas of the PSMA uptake (arrowheads). These findings are highly suggestive of Paget’s disease

A single lesion often favors a benign finding, e.g., fibrous dysplasia, which appears as an expansive well-defined lesion with ground-glass attenuation. Benign lesions can also appear as focal mild medullary uptake without any correlated structural bone lesions on CT. On the other hand, PCa metastasis without CT correspondence appears as moderate to high PSMA focal uptake in the bone marrow, often multiple, which is more common in very aggressive disease when the bone destruction has not been demonstrated yet [35]. Faint and heterogeneous PSMA uptake delineating the cortex of pelvic bones is associated with characteristic morphological changes, such as diffuse sclerosis, thickened cortex, bone expansion, and coarsened trabeculae, and is typical for Paget’s disease. See Fig. 6.

**Fig. 7:**
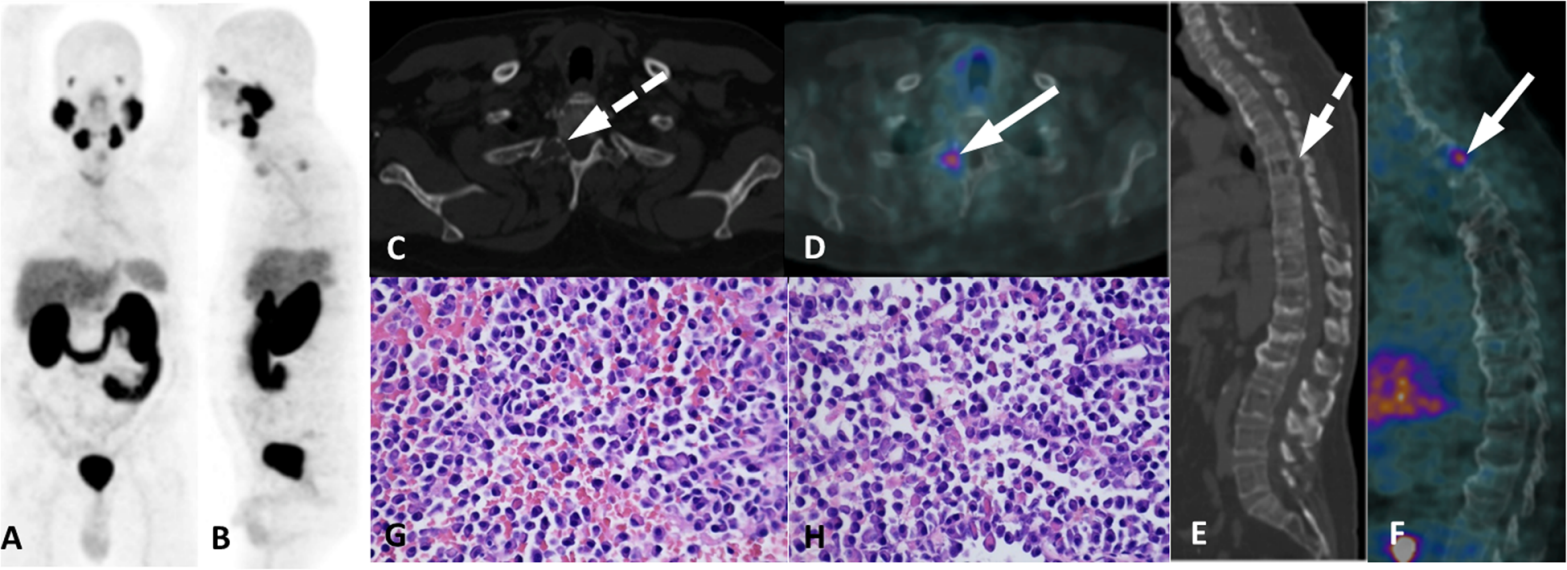
Multiple myeloma with PSMA uptake. ^68^Ga-PSMA PET/CT (**a**, **b**), axial CT (**c**), axial PET/CT (**d**), sagittal (**e**) and sagittal PET/CT (**f**) images show multiple osteolytic lesions (dashed arrows) in the whole axial skeleton but there is a mild uptake in the right pedicle of T2 (arrows), confirmed as multiple myeloma after imaging-guided biopsy (**g**, **h**)

**Fig. 8:**
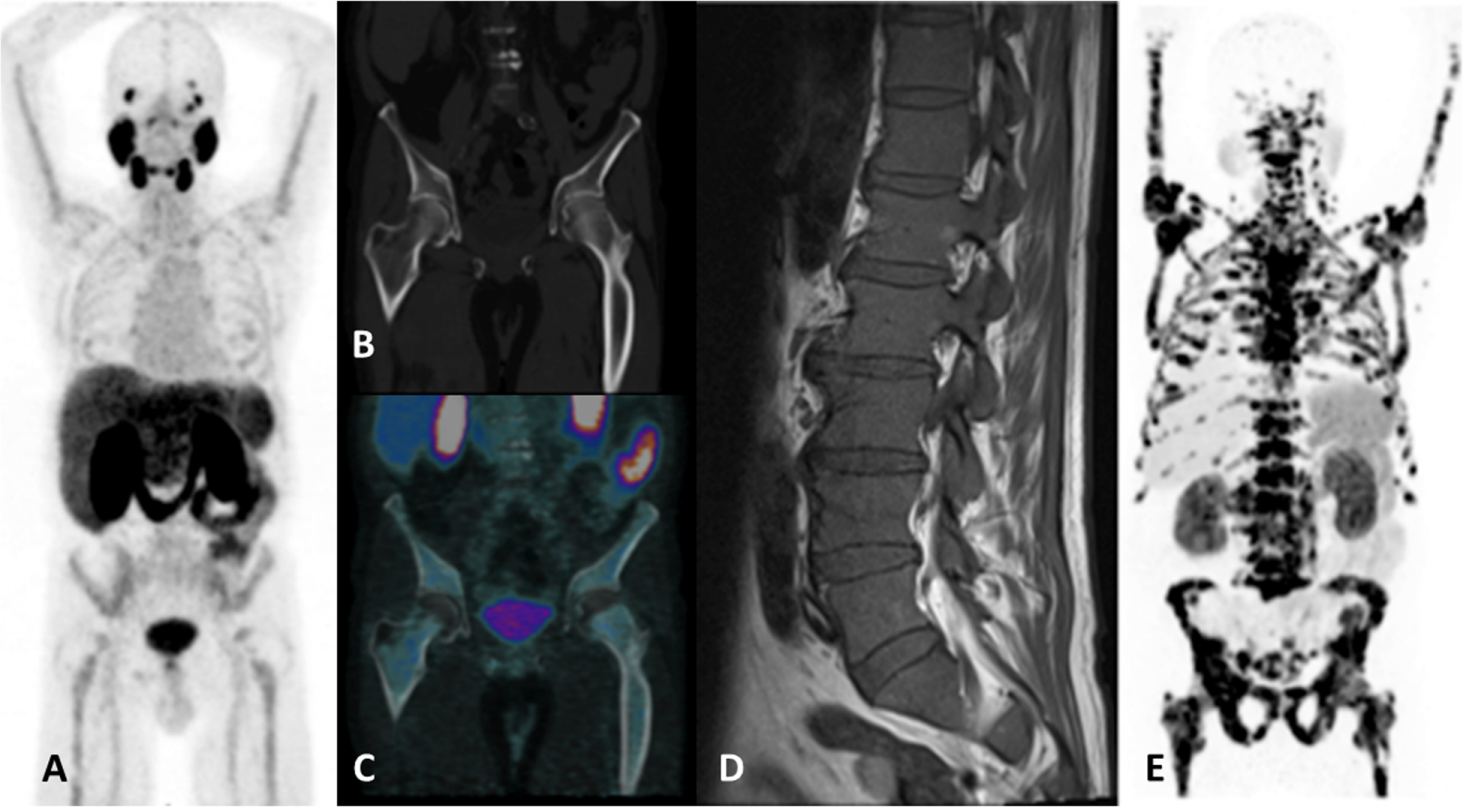
Patterns of diffuse bone PSMA uptake in two different patients. ^68^Ga-PSMA-PET/CT MIP (**a**), coronal CT (**b**) and coronal PET/CT (**d**) images show dense and inhomogeneous bone marrow throughout the skeleton with a discrete diffuse uptake, in a patient with a previous diagnosis of myelodysplasia syndrome. A T1w (**d**) MRI corroborates showing a diffuse infiltration of the bone marrow. A neoplastic infiltration in a different patient is shown on another ^68^Ga-PSMA-PET/CT MIP (**e**) to highlight the uptake pattern differences

Despite the fact that multiple lesions are likely to have a secondary origin, we still need to pay close attention to the morphologic aspects (lytic vs blastic lesions) and notably to other exams (imaging and laboratory findings) in addition to clinical history, as there are known underlying comorbidities (Fig. 7). In contrast, diffuse, faint and homogeneous uptake in the bone marrow is most likely related to an underlying hematological disease rather than to neoplastic infiltration, which more often appears with heterogeneous and intense PSMA uptake (Fig. 8).

**Fig. 9:**
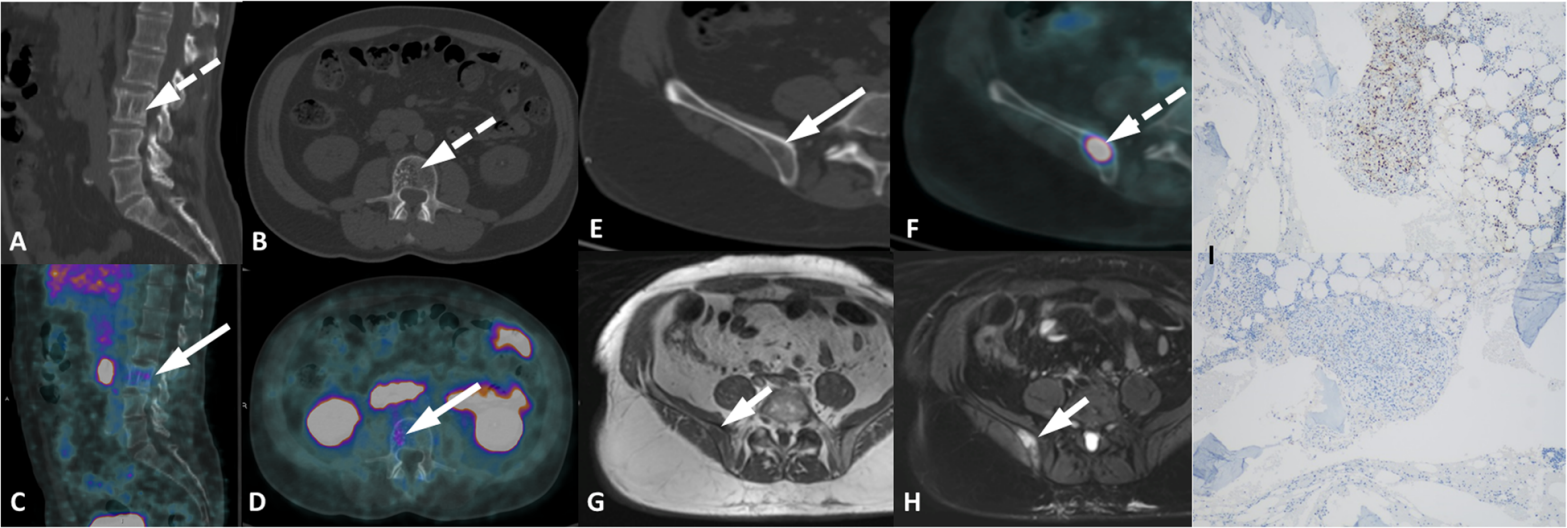
Different patterns of osseous hemangioma in two patients. Patient 1, spinal hemangioma (**a-d**). Sagittal CT (**a**), axial CT (**b**), sagital PET/CT (**c**), e axial PET/CT (**d**) show mild uptake (arrows) in a thickened vertical trabeculae lesion (dashed arrows) surrounded by fat marrow in the right side of L3, typical of hemangioma. Patient 2, iliac hemangioma (**e-i**). Axial CT (**e**) and axial PET/CT (**f**) images showi a lytic lesion (arrow) in the right iliac bone with intense uptake (dashed arrow) and axial T1w (**g**) and T2w Fat Sat (**h**) MR images demonstrated a bone lesion without aggressiveness (short arrows). Bone hemangioma was confirmed with percutaneous biopsy and immunohistochemistry (**i**) showed PSMA expression on the endothelial cells membrane

Although low to moderate PSMA avidity is expected when evaluating nonprostatic bone-related diseases, some lesions might appear with higher uptake, which could lead to an erroneous diagnosis (metastases), particularly in studies without morphological correlation, for which further investigation (MRI and/or biopsy) is mandatory. PSMA uptake in bone hemangiomas is variable, and typical CT appearance is essential for diagnosis. The most common morphological finding is thickened vertical trabeculae lesions surrounded by fat marrow or vascular lacunae presenting a faint PSMA uptake. Occasionally, the morphological aspects on CT are not typical or even absent, and in those cases, proceeding with a different type of morphological exam, such as MRI, would be advised. Hemangiomas with high uptake usually present PSMA expression in the endothelial cells, and when morphological findings are not enough to determine a diagnosis, a biopsy should be the next step. See Fig. 9.

#### Inflammatory and infectious processes

Among various inflammatory and infectious processes, PSMA uptake not related to prostate cancer has already been described in numerous thoracic, abdominal and musculoskeletal conditions. Additionally, an inflammatory PSMA uptake in the prostatic bed might occur following radical prostatectomy, and also after other surgeries.

**Fig. 10:**
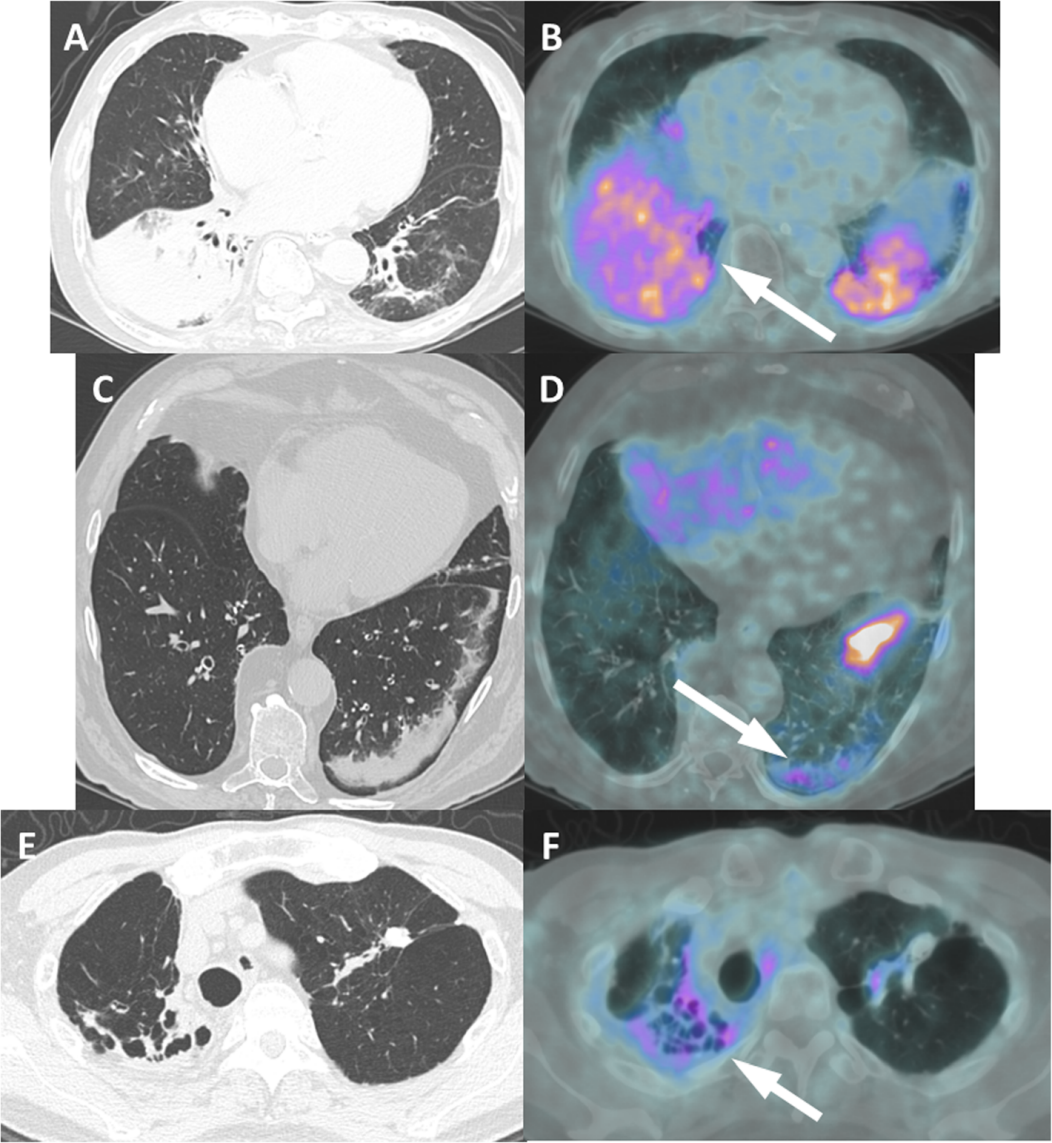
Pulmonary diseases with PSMA uptake. Patient 1, pulmonary consolidation (**a**, **b**). Axial CT (**a**) and PET/CT (**b**) show a pulmonary consolidation with air bronchogram in both lower lobes (arrow on B) of the lungs in a 90-year-old patient presenting cough and fever. Patient 2, pulmonary atelectasis (**c**, **d**). Axial CT (**c**) and PET/CT (**d**) show a laminar atelectasis in both lower lobes of the lungs with tiny PSMA uptake (arrow on D). Patient 3, tuberculosis sequelae (**e**, **f**). Axial CT (**e**) and PET/CT (**f**) show retractile bronchiectasis in the upper bilateral lung lobes with a few calcifications and mild uptake (arrow on F), suggestive of tuberculosis sequelae

**Fig. 11:**
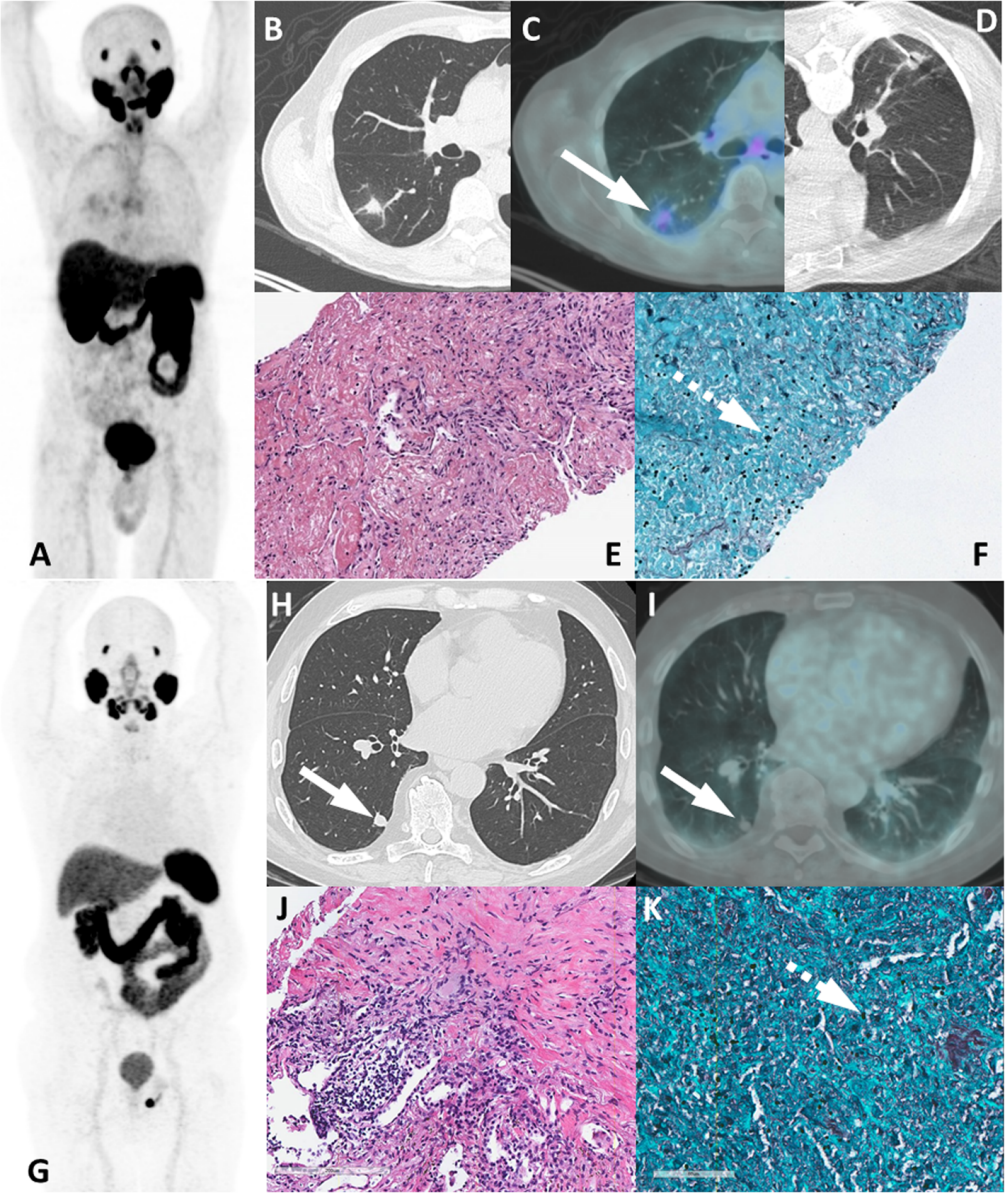
Two different patients with pulmonary histoplasmosis. On top, a 70-year-old patient under staging of prostate cancer (iPSA = 7.0 ng/mL, Gleason 8 = 4 + 4). ^68^Ga-PSMA-PET MIP (**a**), axial CT (**b**), axial PET/CT (**c**) and CT-guided biopsy (**d**) images show an irregular pulmonary nodule with faint uptake (arrow). See also the primary tumor on A (arrowhead). On bottom, a 64-year-old patient with biochemical recurrence (PSA = 0.66 ng/mL, Gleason 9 = 4 + 5) 8 years after radical prostatectomy and under androgen deprivation therapy (Abiraterone) for the last 2 years. ^68^Ga-PSMA-PET MIP (**g**), axial CT (**h**) and PET/CT (**i**) images show a rounded lung nodule in the lower lobe of the right lung without PSMA utpake (arrows). Histology images (**e**, **f**, **j**, **k**) show a positive Grocott stain, with multiple yeasts (dashed arrow) and areas of focal neutrophils conglomerates, consistent with histoplasmosis

The thoracic manifestations of prostate cancer, apart from bone involvement of the vertebral bodies, ribs, sternum, scapulae and clavicles, are mostly confined to the lungs and pleura. Metastases to the lungs are often multiple solid, rounded nodules of varying size that occur along with other metastatic sites (usually skeletal), while the pleural involvement of PCa is represented by diffuse, irregular or nodular thickening of the pleura. On the other hand, inflammatory and infectious processes occur more common in the lungs, are morphologically different and have minimal PSMA uptake. On CT, they range from single solid nodules, often irregular or surrounded by ground glass opacities, to pulmonary consolidations and atelectasis, both associated with reactive lymph nodes in the hilum and mediastinum or bronchiectasis. Interstitial and granulomatous diseases may also present mild PSMA uptake, such as usual interstitial fibrosis, sarcoidosis and tuberculosis. See Fig. 10. The morphological findings on CT allow for differentiation from an eventual and rare form of lymphangitic carcinomatosis related to PCa. Occupational lung diseases such as anthracosis [30] and berylliosis [32] have also exhibited PSMA uptake, which reinforces the need for close examination of the patient’s clinical history. It is important to highlight that these same conditions do not always present PSMA uptake, even in cases of infectious processes such as histoplasmosis (Fig. 11), and the reason might be related to the grade of associated inflammation, which presents a more or less increase in vascular permeability.

**Fig. 12:**
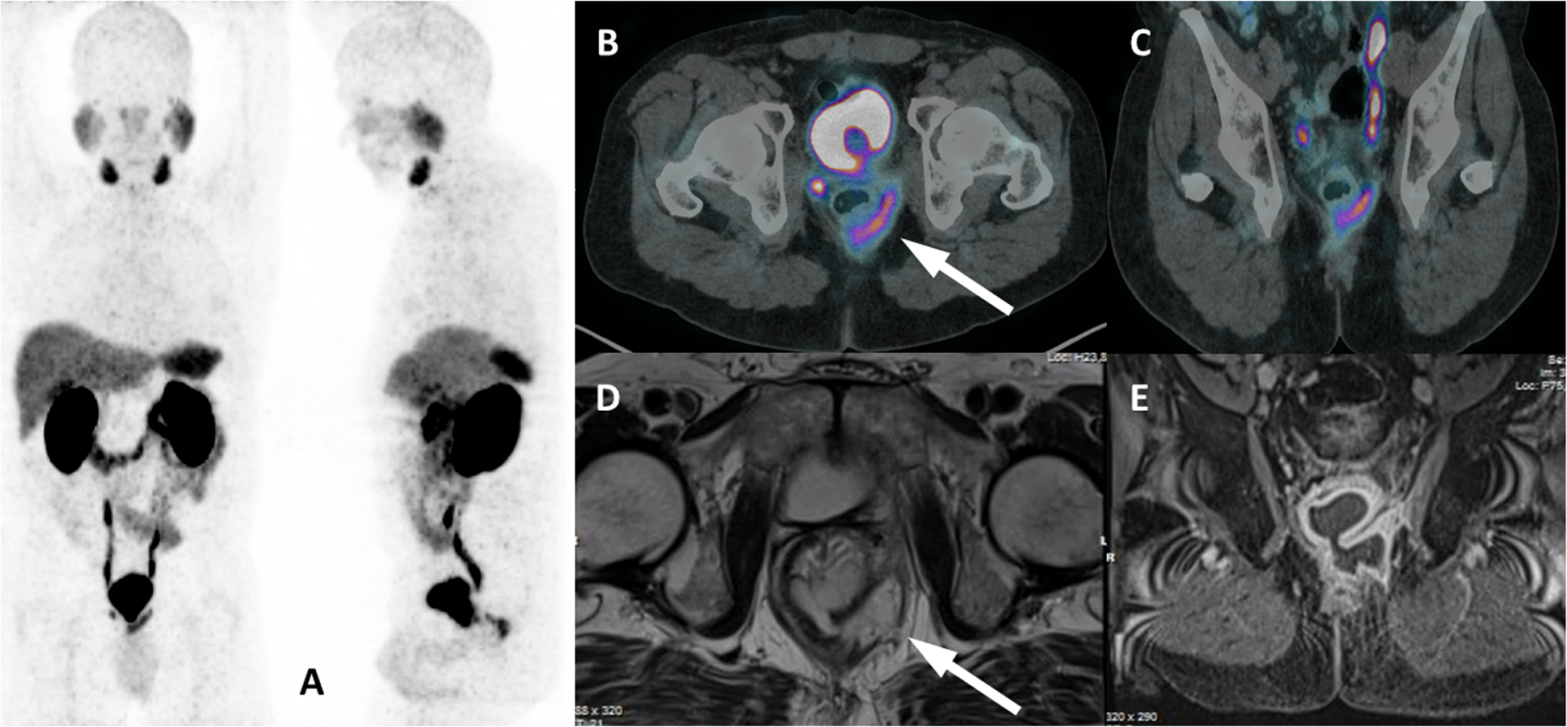
Perianal fistula with PSMA uptake. ^68^Ga-PSMA-PET MIP (**a**), axial PET/CT (**b**), coronal PET/CT (**c**) and axial T2w MR (**d**) and coronal T1w with gadolinum (**e**) show a perirectal fistula in the posterior left ischiorectal fossa (arrows)

**Fig. 13:**
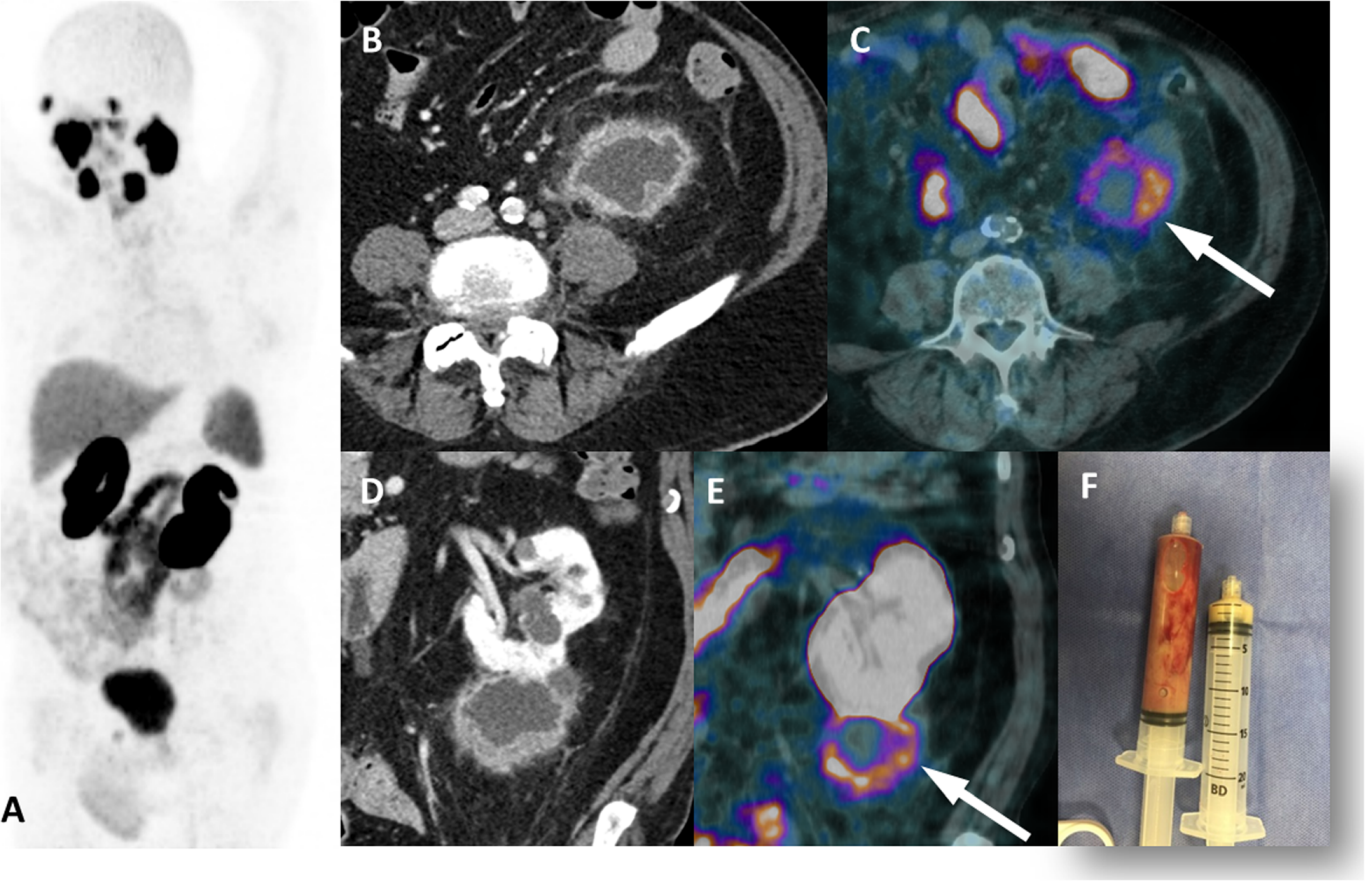
Renal abscess with PSMA uptake. ^68^Ga-PSMA-PET MIP (**a**), axial and coronal contrast-enhanced CT (**b**, **c**), axial and coronal PET/CT (**d**, **e**) images show a complex cystic mass on the lower pole of the left kidney presenting moderate peripheric PSMA uptake (arrows), with purulent content after percutaneous drainage (**f**)

PCa lesions in the abdomen more often involve the liver but may eventually affect the spleen, pancreas, peritoneum, adrenal and the urinary and gastrointestinal tracts [34]. These lesions are frequently multiple and associated with advanced disease stage along with retroperitoneal nodes and osseous metastases. On the other hand, infectious diseases of the abdomen are mostly restricted to one organ and exhibit substantial morphological changes, such as surrounding fat stranding and prominent reactive lymph nodes. Additional MRI may also be requested for further assessment of morphology, especially in cases involving the pelvis (Fig. 12), cases in which multiple treatments were performed, such as surgery and radiotherapy, and cases in which the CT, even with intravenous contrast, is limited to defining the proper anatomy. Sometimes, however, differentiation to a secondary malignancy is difficult, and a biopsy is mandatory for differential diagnosis (Fig. 13).

In the post-operative setting, prostate bed soft tissue uptake can be seen in the scenario of a very recent radical prostatectomy (< 60 days), when clinicians receive the first PSA test after surgery (6–8 weeks). The expected postprostatectomy PSA level is undetectable, or less than 0.05 or 0.1 ng/mL, and when it is greater than 0.2 ng/mL, it can be considered tumor persistence. In this recent postsurgery status, a common morphological change can be seen as fat stranding around the prostate bed (bladder and urethral anastomosis) and the lateral pelvic wall. Prostate bed fat stranding may exhibit mild and ill-defined PSMA uptake that is slightly higher than that in the background but still seen on maximum intensity projection (MIP) images. This soft tissue uptake can be more intense in early PET acquisition (3 min after tracer injection), while there is a high concentration of radiotracer in the blood. The explanation is not completely known, but it might be related to reparative inflammatory processes and/or increased vascularity. Recognition of this uncommon imaging pattern is important because it can be misinterpreted as a local residual tumor.

**Fig. 14:**
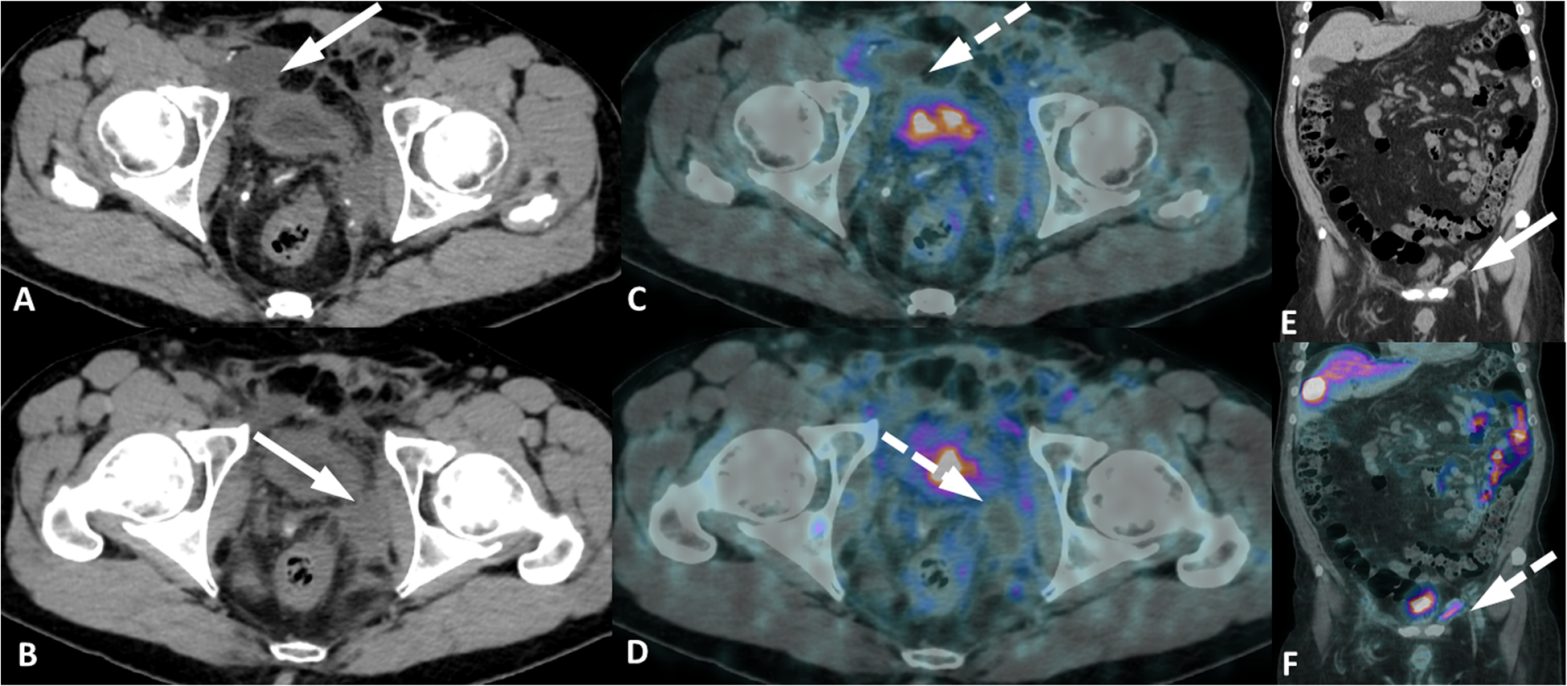
Post-operative inflammatory findings in two patients. Patient 1, prostatic bed inflammation immediate after radical prostatectomy (**a-d**). Axial CT (**a**, **b**) axial PET/CT (**c**, **d**) show faint uptake around bilateral lymphoceles (arrows) and inflammatory changes at bilateral pelvic walls (dashed arrows). Patient 2, inflammatory foreign body reaction of mesh material from left inguinal hernia surgery (**e**, **f**). Coronal CT (**e**) and PET/CT (**f**) images show an elongated hyperattenuating material along the left inguinal canal (arrow) with mild uptake (dashed arrow)

In addition to prostatectomy, another pelvic surgical procedure that may present PSMA uptake is inguinal hernia repair. In most inguinal hernia repairs, a mesh prosthesis is placed along the abdominal wall around the deep or superficial inguinal ring, according to surgical technique [36]. Because there is a foreign body associated with such surgery, early or late inflammatory reactions might occur and are not always symptomatic [37]. Another complication described is mesh retraction, which can present as a hyperattenuating, pseudonodular pattern displaced from the original location and can again be associated with graft inflammatory reactions. These findings can exhibit PSMA uptake, which is expected to be mild and diffuse along the mesh foreign body. See Fig. 14.

Pelvic surgeries are more likely to lead to disease recurrence pitfalls; other surgeries might be easier to differentiate from prostate metastasis, but in all scenarios, clinical information is fundamental to help such distinction. When there is a lack of correlation between clinical information and morphological and molecular PET/CT imaging, further imaging evaluation is recommended. MRI could be the best option for further assessment because of its higher soft tissue contrast resolution than that of CT.

#### Benign neoplasms

Various benign neoplasms present PSMA uptake, and the number of case reports of these conditions is steadily increasing. False-positive tracer uptake has been especially linked to soft tissue lesions, abnormal vascular proliferation, lesions of neurogenic origin, thymoma and adenomas. Most of these lesions could be inferred as not related to PCa due to their location, degree of uptake and clinical and morphological features.

PCa metastases do not commonly affect many of the anatomic sites involved in benign neoplasms, as in the cases of soft tissue lesions or lesions of vascular origin. Soft tissue and musculoskeletal (other than bone) involvement of PCa is unlikely and metastases usually arise in patients with multiple other secondary lesions and high tumor burden. Elastofibroma dorsi, dermatofibroma, acrochordon, fibromatosis desmoid tumor, intramuscular myxoma and pseudoangiomatous stromal hyperplasia of the breast [4, 38] are the main entities reported thus far known to present tracer uptake. The lesions may vary from nodules to large soft tissue masses. Common locations include subcutaneous, muscular, tendon, fascial, palmar, plantar, intra-abdominal, abdominal wall, thoracic wall and the breasts [39, 40].

**Fig. 15:**
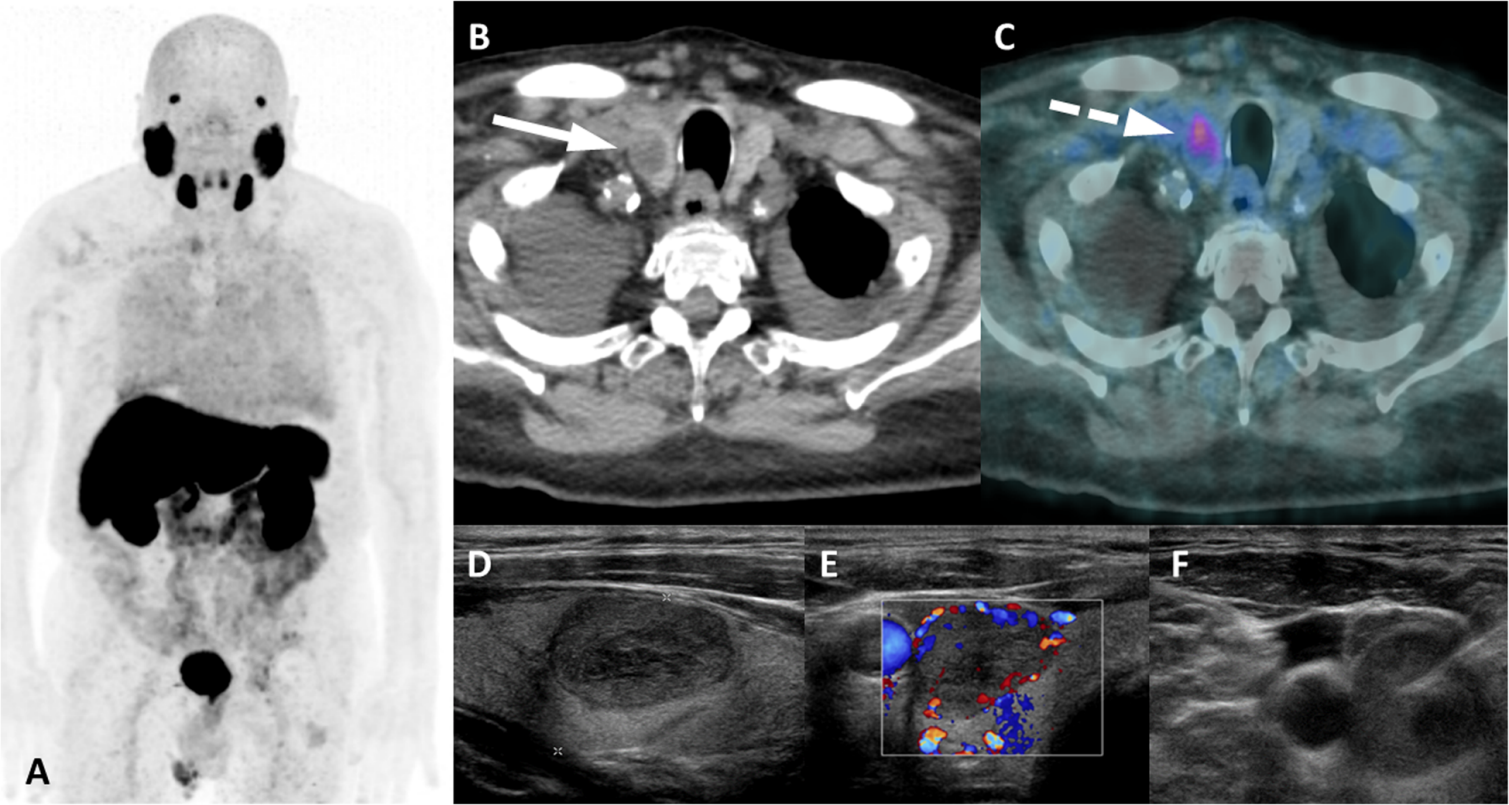
Thyroid benign adenoma with PSMA uptake. ^68^Ga-PSMA-PET MIP (**a**), axial CT (**b**) and axial PET/CT (**c**) images show nodulae with mild uptake (dashed arrow) in the right thyroid lobe (arrow). Thyroid ultrasound revealed a hypoecogenic nodule with cystic areas (**d**) and peripheric vascularization (**d**). Fine needle aspiration of the thyroid nodule (**e**) revealed follicular adenoma without malignant cells

The same rationale can be extrapolated to thymomas and thyroid, parathyroid and adrenal adenomas. Thymomas have typical location – mostly well defined, solid, typically rounded, smooth tumors, with a bosselated surface in the anterior mediastinum [41–43].. Thyroid and parathyroid adenomas are readily recognized as thyroid nodules or focal areas of uptake adjacent to the thyroid gland [44]. See Fig. 15. Adrenal adenomas may appear as enlarged adrenals (uni- or bilateral), as a low-density adrenal nodule with attenuation < 10 HU or as an adrenal mass (11). Those entities seldom present challenges to an aware reading physician due to their high prevalence, typical tomographic features and uncommon location for PCa metastases.

**Fig. 16:**
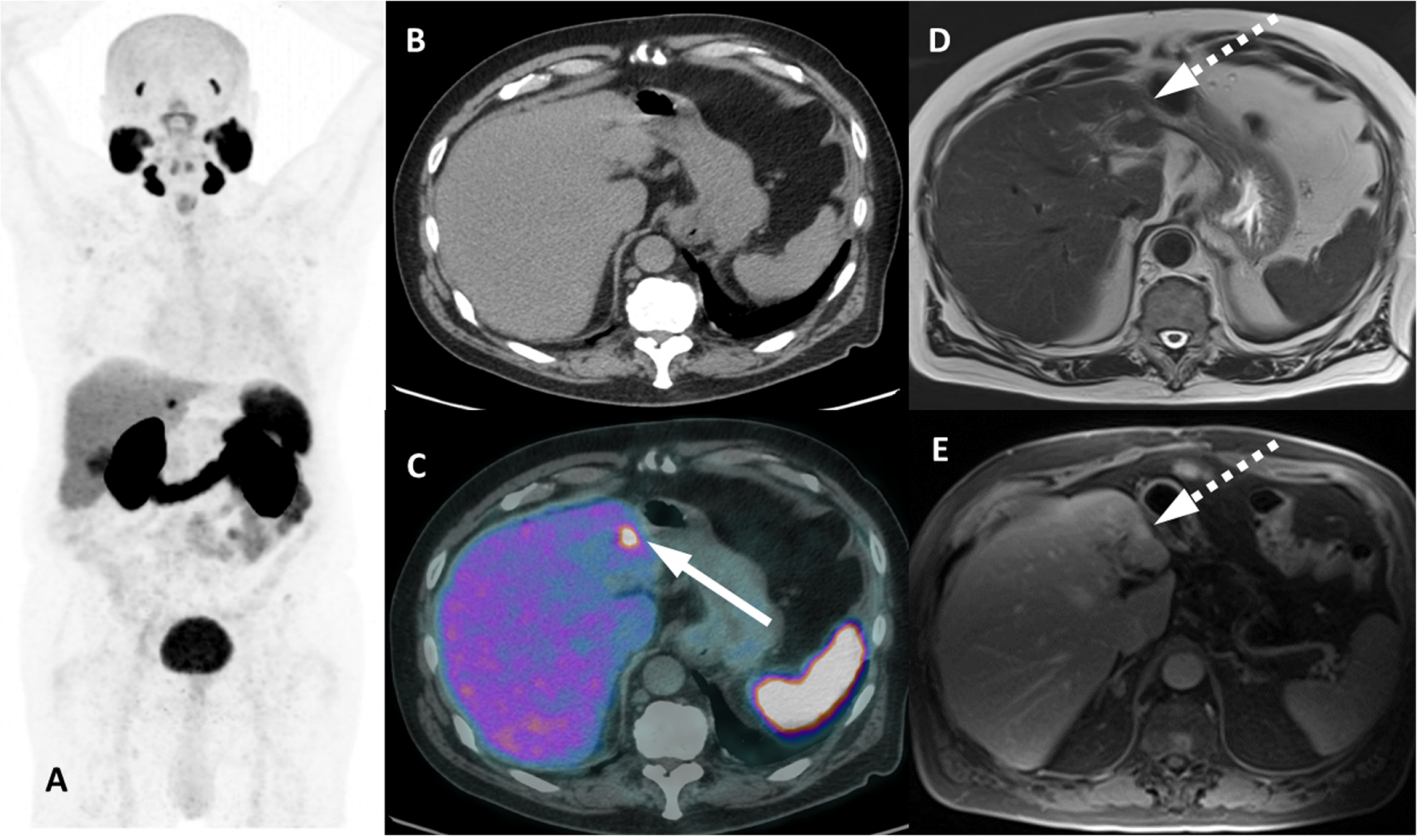
Benign hepatic hemangioma with PSMA uptake. ^68^Ga-PSMA-PET MIP (**a**) and axial PET/CT (**c**) images show an isolated moderate focal uptake on the left liver lobe (arrow) not seen on non-contrast enhanced CT in (**b**), which was subsequently caractherized as a typical hemangioma (dashed arrows) in axial T2w (**d**) and axial T1w after gadolinum (**e**). Contrast-enhanced CT performed 10 years earlier (not shown) had already characterized the lesion

Regarding the level of PSMA expression, most of benign lesions present lower avidity than secondary lesions do, although in a few cases some lesions may present unexpectedly high expression of PSMA. Exceptions to the rule might be found in lesions of vascular origin, such as hemangiopericytoma [45, 46], angiolipoma [47] and hemangiomas [4, 48, 49]. PET findings may be deceptive and tricky and potentially lead to misdiagnosis. The most common presentation is of nodular lesions, mostly of small dimensions, potentially encountered in multiple locations throughout the body [47, 50]. Hemangiomas are especially worth highlighting, because interpretation of PSMA PET findings may be challenging since they may present intense tracer uptake [48, 49, 51–53]. Typical imaging features include well-defined lesions with low attenuation and slow centripetal enhancement following intravenous contrast administration. Larger lesions may fill with the contrast agent more slowly and heterogeneously than smaller lesions do. Given the dynamic nature of the enhancement pattern, a triphasic contrast-enhanced CT within the PET/CT study may be requested. Common sites of involvement include the liver and the spleen. In both organs, hemangiomas rank among the most prevalent benign lesions, so that lesions presenting PSMA uptake in these organs should have its nature questioned. When findings are equivocal, subsequent evaluation by contrast-enhanced MRI studies may be suggested to aid in clarification (Fig. 16).

**Fig. 17:**
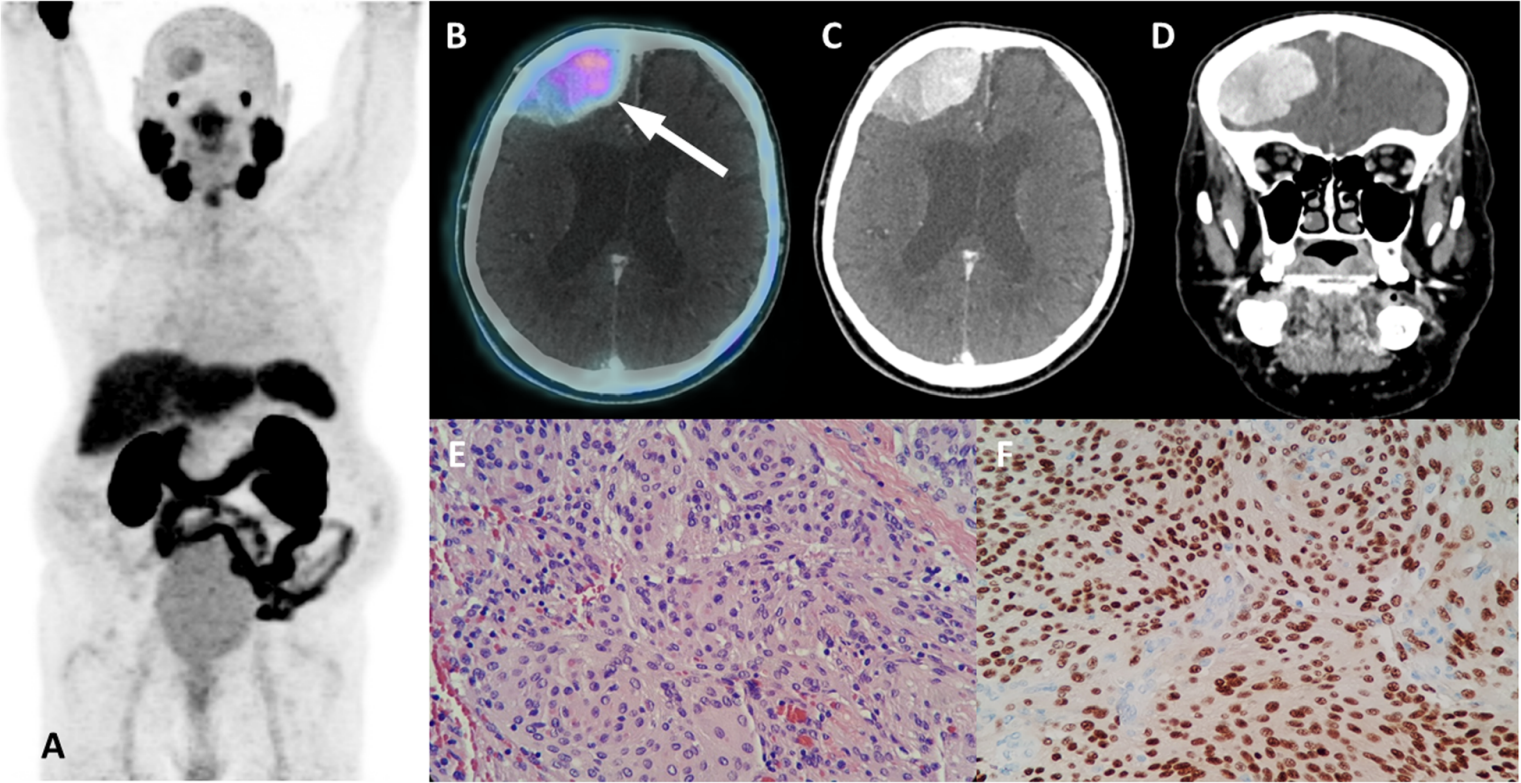
Typical meningioma with PSMA uptake. ^68^Ga-PSMA PET MIP (**a**), axial PET/CT (**b**) and axial and coronal contrast-enhanced CT (**c**, **d**) images show right frontal extra-axial vascularized lesion with dural tail (arrow), presenting mild uptake. Histopathology and immunmohistochemistry images (**e**, **f**) from tumor ressection depicted meningothelial cells with high progesterone stainning, confirming meningioma

**Fig. 18:**
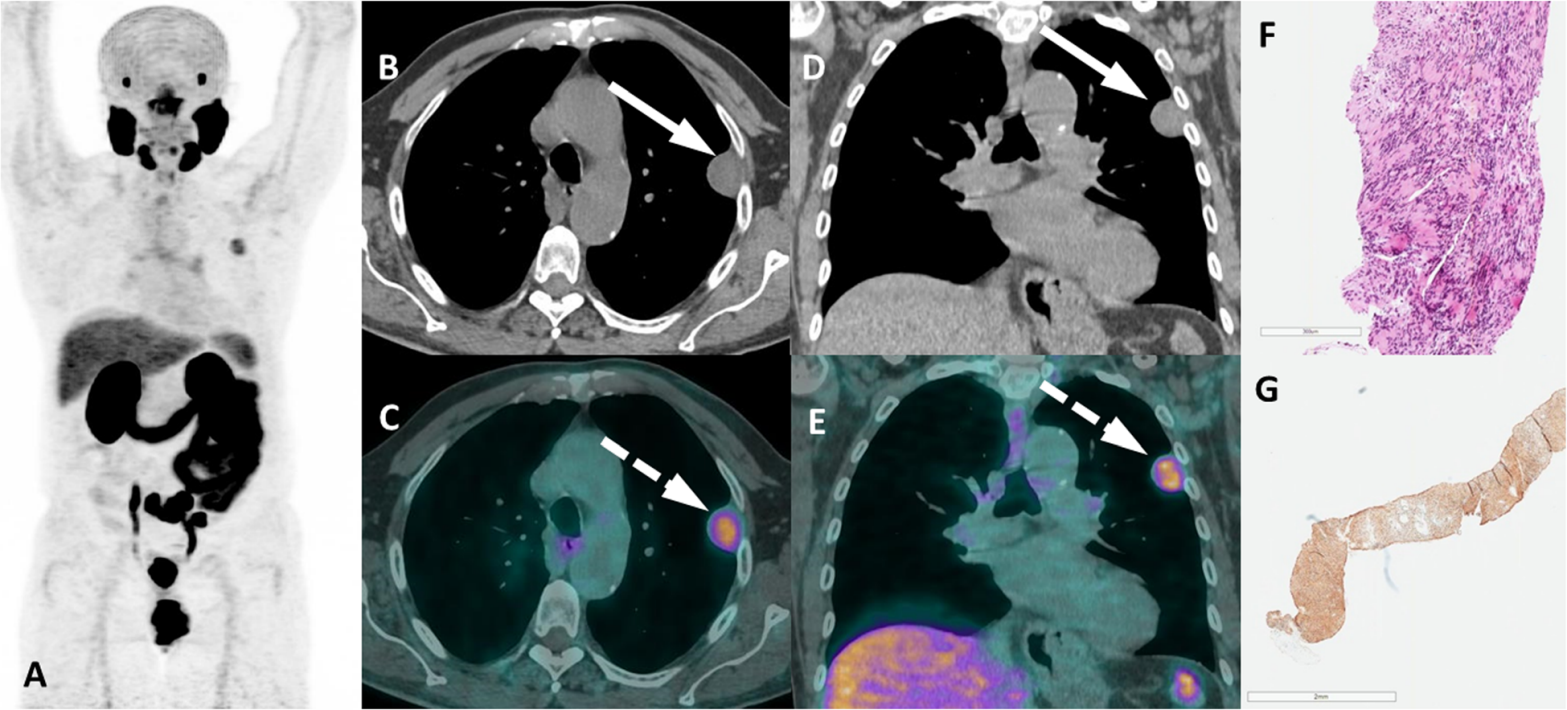
Intercostal schwanomma with PSMA uptake. ^68^Ga-PSMA PET MIP (**a**), axial and coronal CT (**b**, **d**) and PET/CT (**c**, **e**) show extrapulmonar intercostal lesion (arrows) with moderate uptake (dashed arrows), which was confirmed as a schwannoma by percutaneous biopsy. Histopathology showing spindled Schwann cells (**f**) with positivity S100 immunohistochemistry staining (**g**)

A group of entities with variable location and degree of uptake are lesions of neurogenic origin. This category includes meningiomas, schwannomas, peripheral nerve sheath tumors and neurofibromas. Meningiomas may appear in any structure lined by meninges adhered to the internal dural layer. Most lesions are well defined, peripherally located, dural based, slightly hyperdense or isodense to normal brain tissue, and may have calcification. There is usually bright and homogenous enhancement of the lesion following the administration of contrast agent. Large lesions might promote adjacent edema or neighbor important brain structures leading to the onset of neurologic symptoms (Fig. 17). In turn, schwannomas may affect intracranial structures, the spine and spinal nerve roots, the trunk (intercostal nerves, the mediastinum, the retroperitoneum, and the gastrointestinal tract) and the limbs (especially flexor surfaces, such as ulnar and peroneal nerves) (Fig. 18). On CT, schwannomas usually appear as well circumscribed nodules or masses of low to intermediate attenuation. Commonly, they dislocate neighboring structures, such as bone, with no signs of invasion, frequently with smooth, corticated edges due to remodeling. They characteristically grow in close proximity to nerves, often along the neuroforamen [54–56]. Finally, neurofibromas are benign peripheral nerve sheath tumors strongly associated with neurofibromatosis type 1. They appear as well-defined hypodense nodules mostly in subcutaneous tissues, although they may affect almost the entire body. Clinical and morphological features are meaningful for the differential diagnosis of lesions of neurogenic origin, and correlation with MRI may be elucidative in doubtful cases or simply for confirmation purposes.

#### Malignant neoplasms

Knowledge of PCa behavior, including typical and atypical disease appearances, was deeply influenced by the emergence of PSMA PET imaging, leading to previously non-imagined imaging features of PCa [34]. Additionally, with the wide adoption of PSMA PET for PCa, more patients are prone to harboring concomitant malignant neoplasms (by age, genetic or even access selection) and are undergoing highly detailed whole-body examinations, such as PET/CT or PET/MRI, leading to an expected increase in the detection of concomitant nonprostatic malignancies in these patients. As expected, the body of literature available on this topic is rapidly growing, based mainly on case reports of false-positive findings [4, 57–61]. Synchronic malignant neoplasia has been described as rarer and showed lower PSMA uptake than did atypical PCa metastasis and benign lesions in a large cohort of PCa patients who underwent PSMA PET/CT imaging [62]. However, another large retrospective series with 1889 patients specifically compared lung lesions detected on PSMA PET/CT imaging with presumptive or histological diagnosis, showing that the degree of uptake measured by SUVmax could not differentiate between PCa metastasis and lung cancer [31].

PSMA expression by nonprostatic tumors has been shown by previous immunohistochemistry studies [14, 63–65]. In this context, renal cell carcinoma (RCC) is one of the first and most assessed tumors by PSMA PET/CT imaging in the literature because it is a heavily vascularized tumor with well-documented PSMA expression in the neovasculature, as shown by immunohistochemistry and imaging studies [64, 66]. However, the emergence and rapid worldwide adoption of PSMA PET imaging for PCa has produced many anecdotal reports of unintentional and potential confounding or false-positive PSMA ligand uptake in several cancers, such as lung, colorectal, gastric, pancreatic, and thyroid cancers, as well as sarcomas, lymphomas and other tumors [2].

**Fig. 19:**
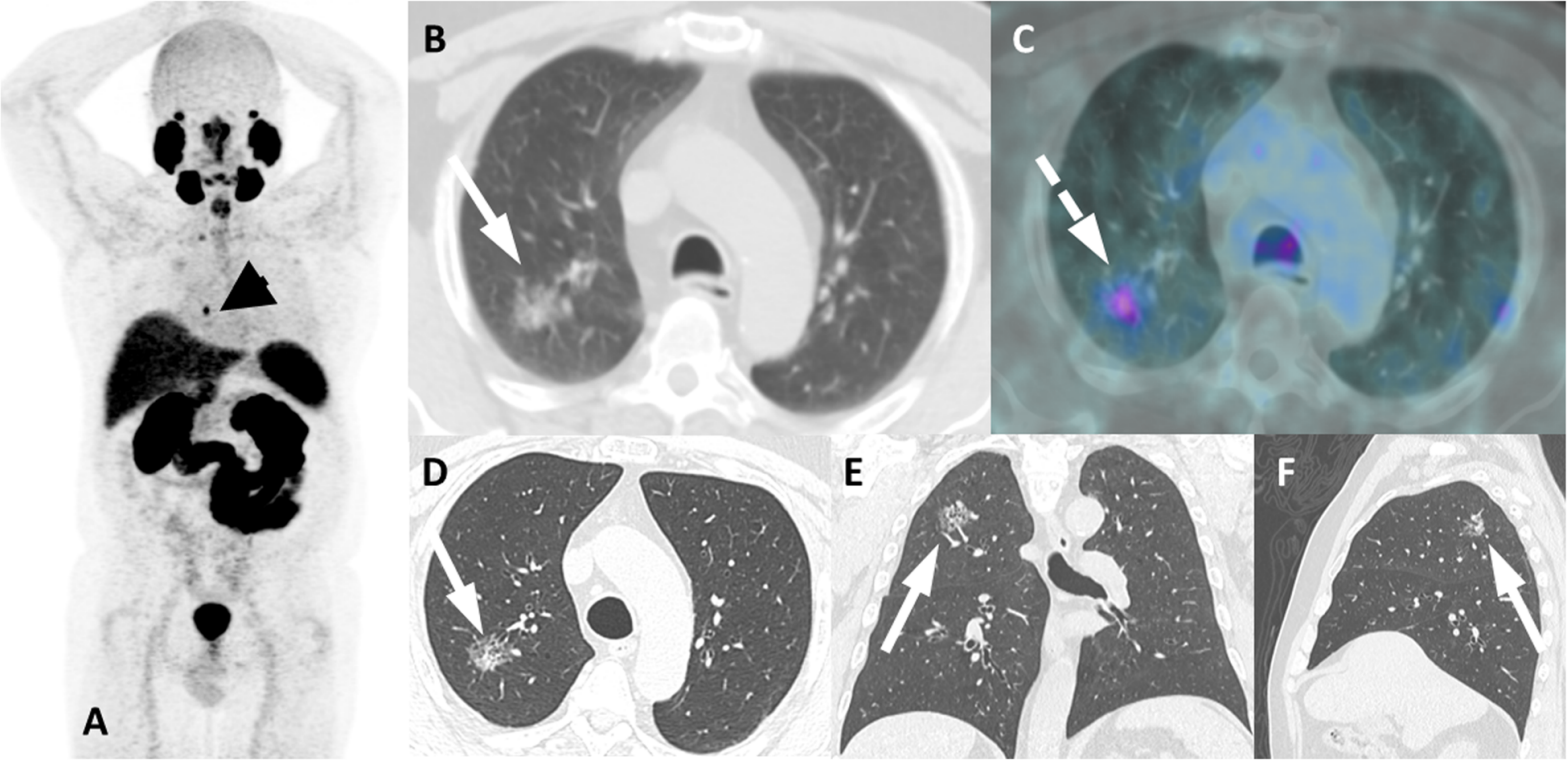
Lung adenocarcinoma with PSMA uptake. ^68^Ga-PSMA-PET MIP (**a**), axial CT (**b**) and axial PET/CT (**c**) and axial, coronal and sagittal dedicated lung CT (**d**, **e** and **f**) show a slight uptake (dashed arrow) in peribronchial subsolid pulmonary nodule in the posterior segment of the right upper lobe (arrows), which biopsy revealed primary lung adenocarcinoma. Note also a confirmed prostate cancer metastatic right pulmonary hilar lymph node with intense uptake (arrowhead in A)

In concordance with the authors’ experience and as naturally expected, more prevalent neoplasms in men are responsible for the most common findings of nonprostate malignancies in PSMA PET imaging. Lung cancer can produce challenging findings and has been the subject of some studies. The CT presentation of lesions is an important (not rarely enlightening) step in analysis, and features such as the number, the size, the presence of spiculated (an important marker of suspicion for primary malignancy) or smooth borders and the presence of ground-glass components can be valuable for interpretation (Fig. 19). In a scenario of nonmetastatic or non-advanced metastatic PCa, a lung lesion that may alter patient management should be clarified by biopsy, regardless of the degree of PSMA ligand uptake. This approach can also be helpful for nonpulmonary equivocal lesions**.**

Central nervous system (CNS) primary tumors (gliomas) may also overexpress PSMA [2]. Moreover, there is a significant difference in PSMA expression between low and high-grade gliomas, explained by the difference in their neovasculature [67]. Isolated CNS PCa metastases are extremely uncommon, so correlation with current disease staging while facing a brain lesion on PSMA is mandatory to consider further investigation and exclude a primary tumor that are often more aggressive than PCa.

Colorectal cancer is another condition in which PSMA expression has been reported [59, 60]. Specifically, for pelvic lesions, in our experience, the degree of uptake and CT pattern showing wall thickening or colon-based lesions can be helpful in distinguishing equivocal lesions from prostate atypical metastases or direct rectal invasion by locally advanced PCa [34]. A particularly confounding site is the liver, a frequent site of colorectal metastasis, where PCa metastasis can exhibit reduced expression of PSMA, reducing the specificity. A valuable clue is that PCa metastasis to the liver is infrequent in nonadvanced stages of disease; therefore, hepatic lesions in this setting must be submitted to additional diagnostic workups, such as dedicated MRI, because even primary hepatic neoplasms can also exhibit PSMA expression [68].

**Fig. 20:**
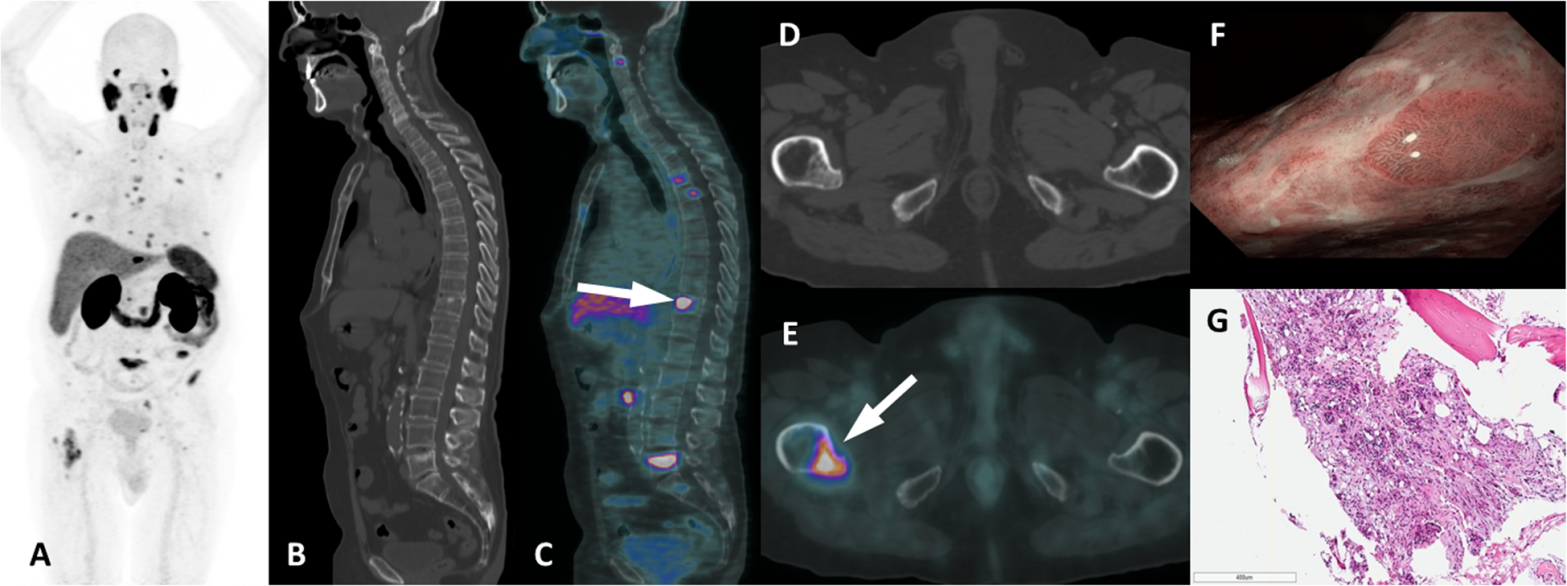
Signet-cell gastric adenocarcinoma with bone metastases in a 82-year-old patient with prostate cancer 5 years after radical prostatectomy and biochemical recurrence (PSA = 0.67 ng/mL). ^68^Ga-PSMA-PET/CT MIP (**a**), coronal and axial CT (**b**, **d**) and coronal and axial PET/CT (**c**, **e**) images show multiple osteoblastic lesions with intense uptake (arrows in C and E). The patient performed an endoscopy (**f**) due to dyspeptic symptoms and diagnosed a signet cell gastric carcinoma. Additionally, the discrepancy between low PSA level and high tumor burden on PSMA PET imaging, patient underwent a left femur percutaneous biopsy that histopathology confirmed metastatic adenocarcinoma from gastric origin (**g**)

**Fig. 21:**
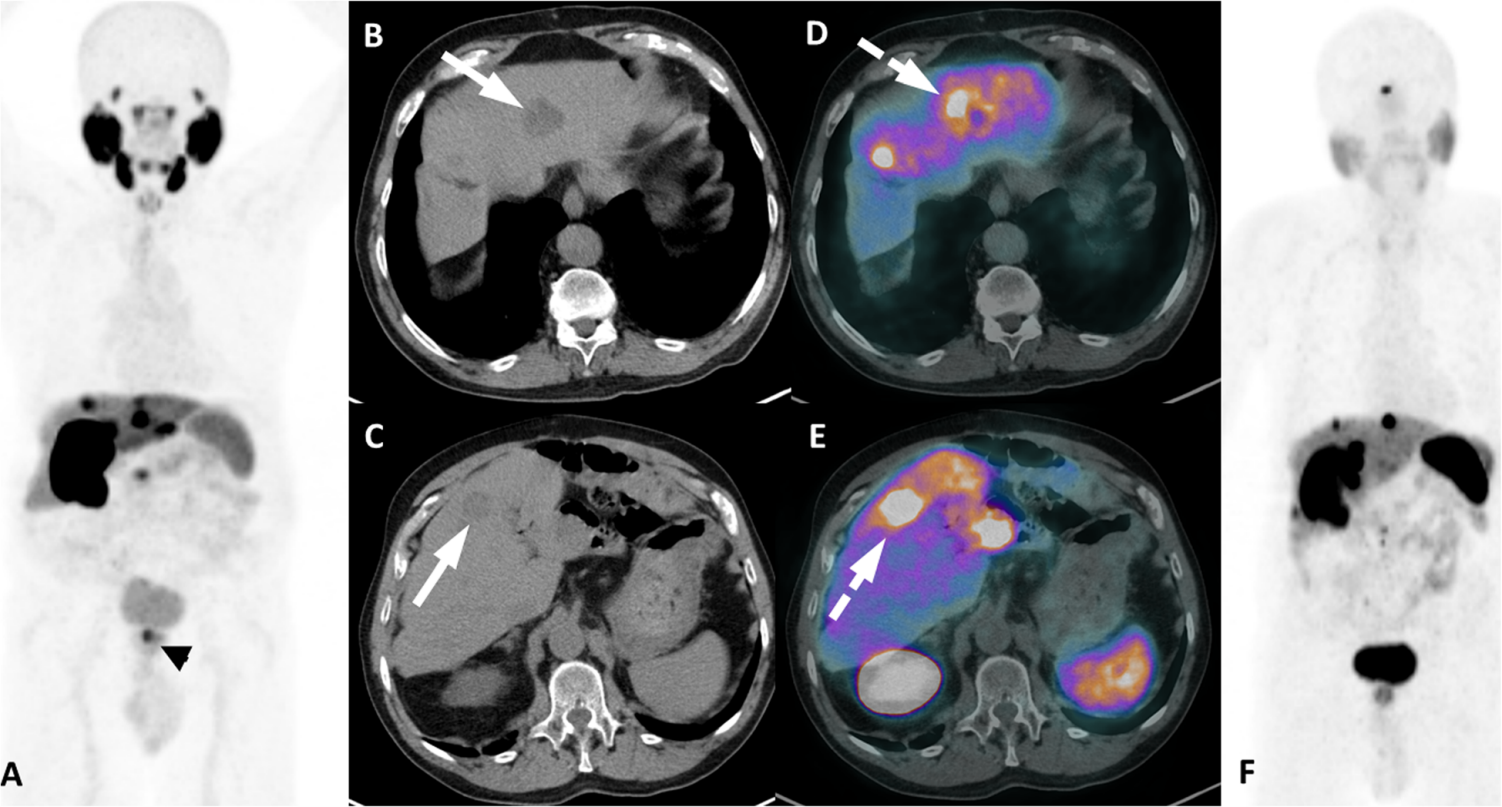
Neuroendocrine liver metastases in ^68^Ga-PSMA and ^68^Ga-DOTATATE PET. ^68^Ga-PSMA PET MIP (**a**), axial CT (**b**, **c**) and axial PET/CT (**d**, **e**) show a high uptake (dashed arrows) in metastatic liver lesions of neuroendocrine tumor (arrows). Note also the primary prostate tumor (arrowhead in A). ^68^Ga-DOTATATE PET MIP (**f**) 2 years before showing that these hepatic lesions had also high somatostatin receptors expression

Gastric and pancreatic cancers are other sources of potential PSMA-expressing lesions because these tumors also have documented high expression of PSMA in their neovasculature [69] (Fig. 20). Because these organs are very atypical sites of PCa metastasis, one should automatically think in synchronicity when finding a gastric or pancreatic lesion. For pancreatic tail lesions, neuroendocrine etiology can be suspected, and in some doubtful situations, ^68^GA-DOTATATE PET/CT or PET/RM can provide a resolute evaluation (Fig. 21).

**Fig. 22:**
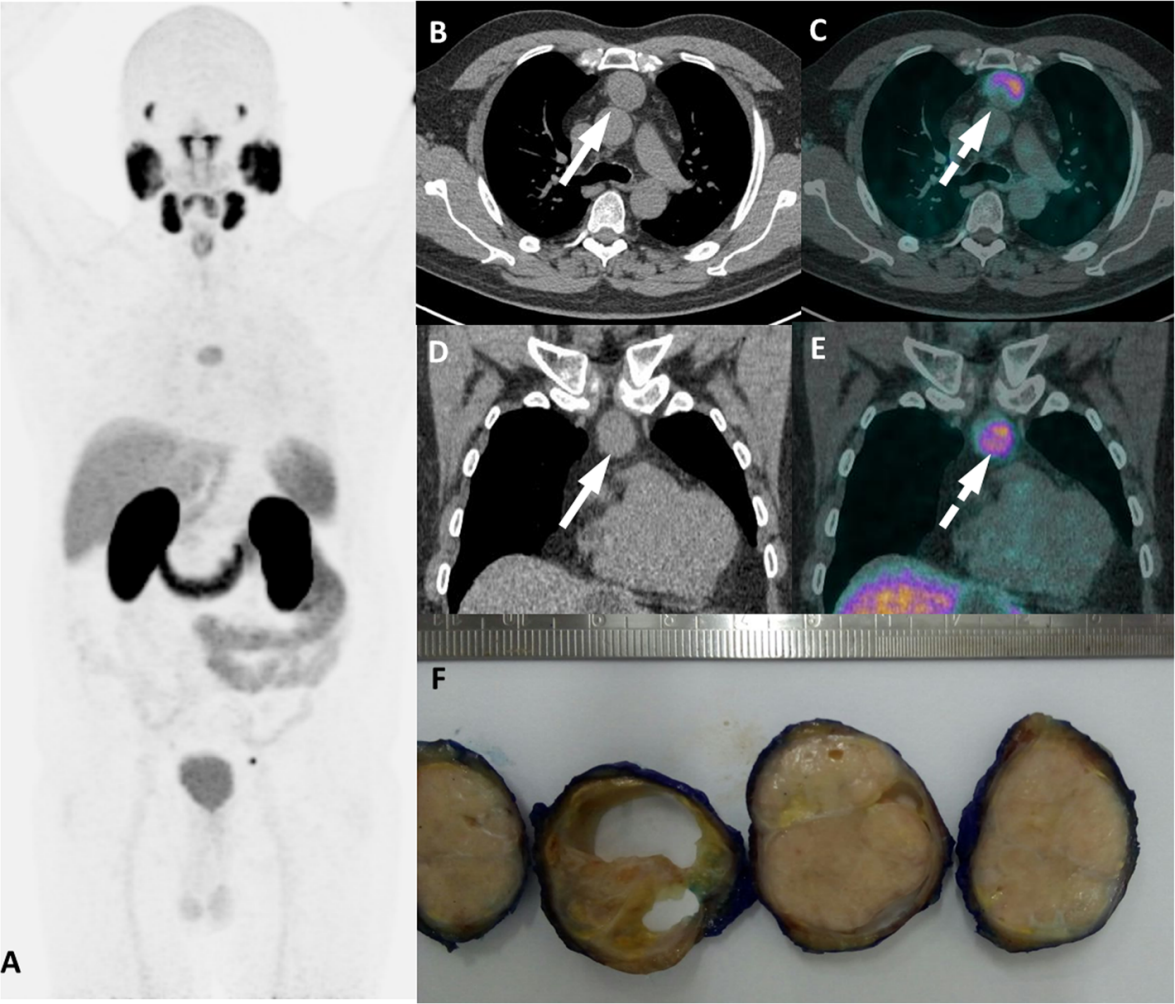
Thymic carcinoma with PSMA uptake. ^68^Ga-PSMA-PET/CT MIP (**a**), axial and coronal CT (**b**, **d**) and axial and coronal PET/CT (**c**, **e**) images show an anterior mediastinal mass (arrows) with mild uptake (dashed arrows). As an atypical spread for prostate cancer, this lesion was first biopsied and then resected diagnosed as thymoma as shown in pathology macroscopy (**f**). *Case courtesy of Bernardo Bacelar, MD and Thiago F. Nunes, MD*

**Fig. 23:**
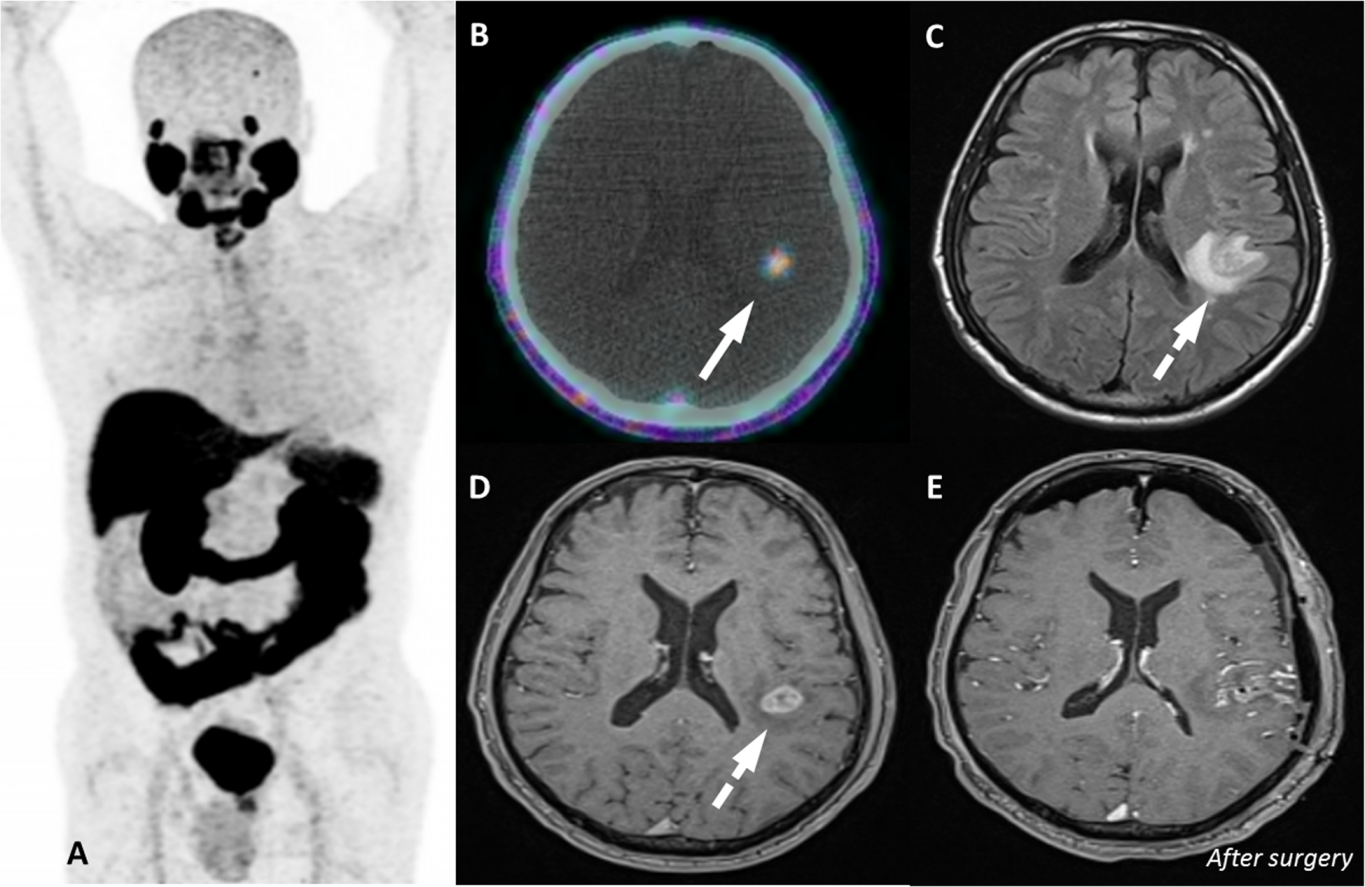
Glioblastoma multiforme with PSMA uptake. ^68^Ga-PSMA PET MIP (**a**), axial PET/CT (**b**), axial FLAIR (**c**) and T1w after gadolinium (**d**) images show a mild uptake in a cortico-subcortical lesion on the left parietal lobe (arrows) corresponding to a necrotic lesion with anelar enhancement and adjacent vasogenic edema (dashed arrows), confirmed as a Glioblastoma after surgical resection (**e**)

Several other malignant neoplasms can exhibit PSMA ligand uptake on PET imaging [4, 70], and the above-mentioned principles of interpretation and contextualization of the imaging findings will help reading physicians to suspect nonprostatic lesions or to recommend further appropriate investigations to better assess lesion etiology (Figs. 22 and 23).

### Perspectives – from immunobiological knowledge and imaging pitfalls to potential clinical usage

**Fig. 24:**
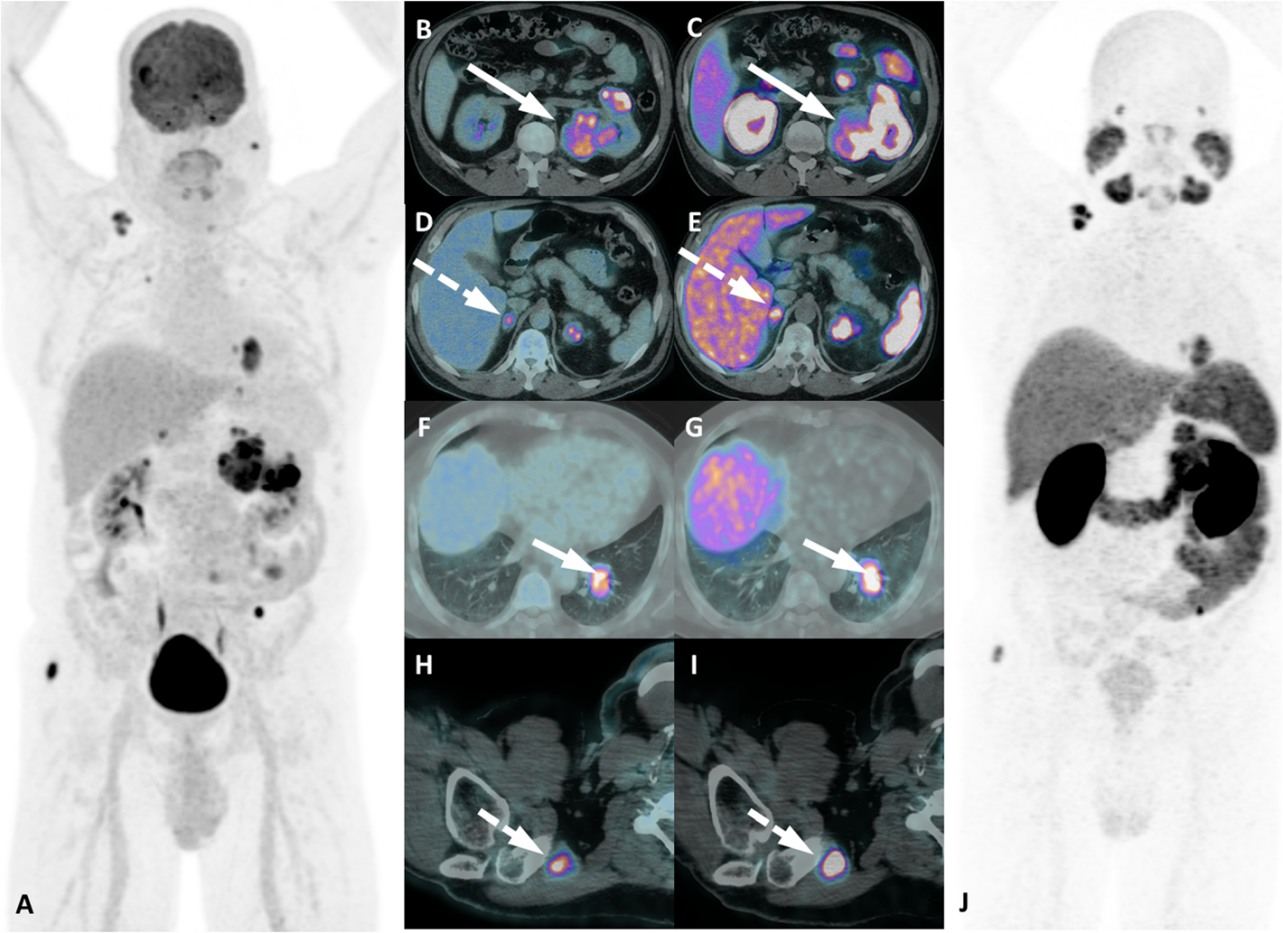
Metastatic clear renal cell carcinoma staging with ^18^F-FDG and ^68^Ga-PSMA-PET/CT. ^18^F-FDG-PET/CT MIP (**a**) and axial PET/CT at different levels (**b**, **d**, **f** and **h**) and ^68^Ga-PSMA-PET/CT MIP (J), axial PET/CT (**c**, **e**, **g** and **i**) images show the high uptake of both tracers in all malignant lesions, highlighting the primary kidney lesion (long arrows), adrenals (long dashed arrows), lung (small arrows) and a soft tissue metastatic lesions (small dashed arrows)

As a new imaging modality, PSMA PET has shown emerging pitfalls in the medical literature. Such pitfalls may serve as an initial disadvantage that can be turned in an opportunity, since new conditions (especially nonprostatic tumors) can be imaged and perhaps in the future might even be considered for PSMA ligand radiopharmaceuticals in a theranostic approach, which is increasingly being used for PCa [71]. Additionally, previous immunobiological knowledge about PSMA expression in nonprostatic disease (particularly in malignant neoplasms) can now be applied and magnified by an in vivo evaluation in a highly sensitive, whole-body imaging modality. Thus, some promising studies have addressed the diagnostic (and therapeutic) potential of PSMA ligand radiopharmaceuticals in non-PCa malignant neoplasms. RCC is the most studied neoplasm within this new context of an “intentional” use of PSMA ligands, since RCC was one of the first recognized neoplasms with high PSMA expression in endothelial tumor neovasculature (Fig. 24). A pilot study prospectively compared the PSMA PET and CT findings of 10 patients (36 lesions) with metastatic RCC, showing not only higher sensitivity of PSMA PET (92 vs 69%) but also changes in intended management [72].

**Fig. 25:**
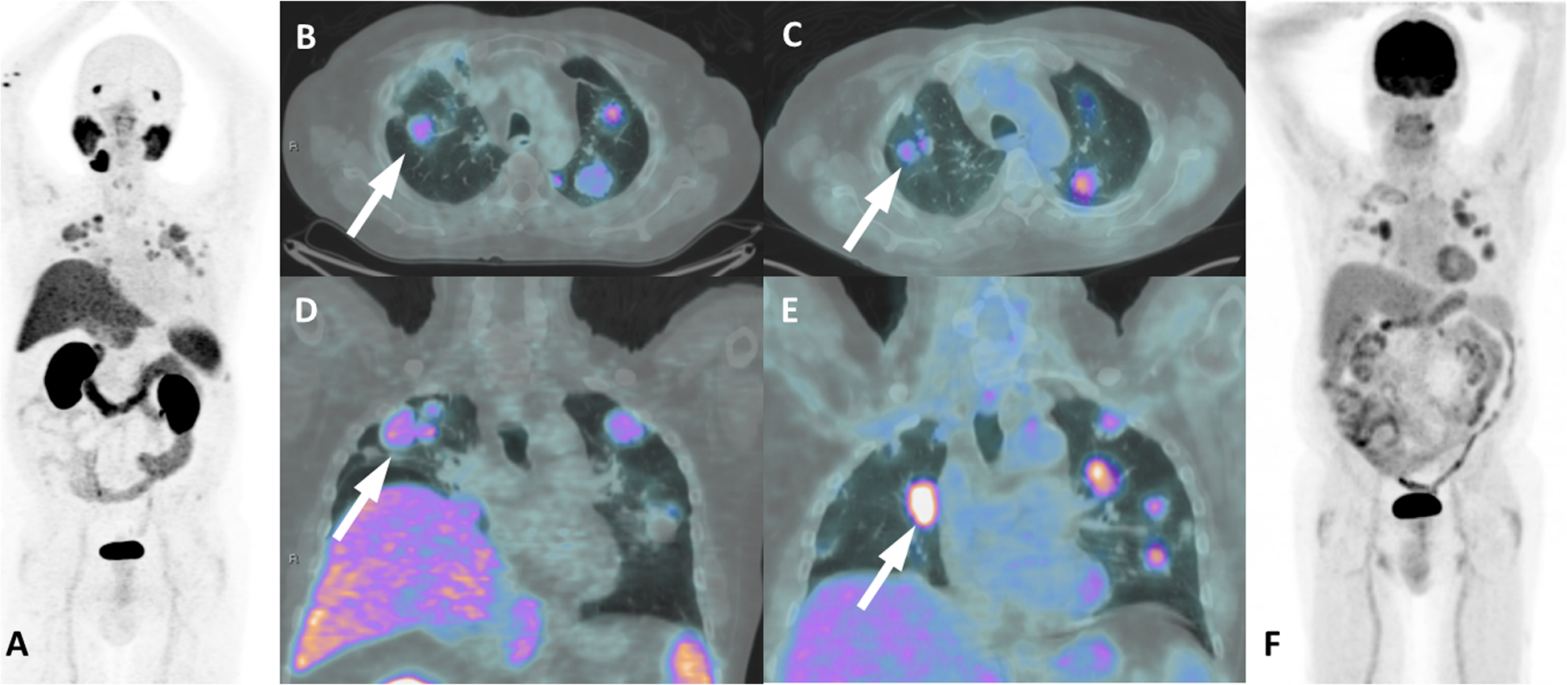
Metastatic adenoid cystic carcinoma in ^68^Ga-PSMA and ^18^F-FDG-PET/CT. ^68^Ga-PSMA-PET/CT MIP (**a**), axial and coronal PET/CT (**b**, **d**) and ^18^F-FDG-PET/CT MIP (**f**), axial and coronal PET/CT (**c**, **e**) images show multiple solid nodules (arrows) with a heterogeneous moderate to intense uptake of both tracers. This patient was referred to treatment with Lu-PSMA after progressive disease despite all previous treatments

Salivary gland tumors are another good example in which a physiological characteristic of PSMA ligand uptake was explored to develop a potential clinical use of the technique. A preliminary study of advanced salivary adenoid cystic carcinomas showed promising results in 10 patients, including high concordance with [^18^F] fluorodeoxyglucose (FDG)-PET/CT findings and immunoexpression of PSMA on tumor cells themselves (luminal staining showed a highly overregulated profile of PSMA expression that is rarely seen in non-PCa tumors) [73]. Linked to this, a clinical trial (NCT 03319641 – clinicaltrials.gov) tried to form a possible rationale for therapeutic applications in adenoid cystic carcinoma by evaluating the uptake of ^68^Ga-PSMA on PET/CT scans. In our experience, PSMA PET/CT has shown good performance, even in comparison with ^18^FDG PET/CT when evaluating adenoid cystic carcinoma of the salivary glands (Fig. 25).

Following the same rationale, diseases in which other modalities (including ^18^FDG-PET/CT) have left some unmet needs are being studied for potential indications for PSMA-ligand PET imaging (and even with theranostic intention). Examples include breast cancer (especially the lobular subtype), thyroid cancer, pancreatic cancer, brain tumors and others [65, 74].

## Conclusions

Nonprostatic diseases exhibiting PSMA uptake on PET are becoming more common as the number of performed scans increases. The first step to differentiate them from metastatic PCa is recognizing the variety of conditions presenting PSMA uptake on PET imaging, from inflammatory processes and benign tumors to bone lesions and malignant neoplasms. Understanding the biology behind PSMA uptake represents another primer that favors this distinction. PSMA expression in the apical membrane, which is a marker of PCa cells, presents greater tracer uptake on PET imaging than the level of expression shown in the cytoplasm, which is more often observed in nonprostatic diseases. However, this finding does not enable a clear differentiation between PCa lesions and nonprostatic diseases. Several other characteristics should be considered, such as the pattern of disease spread, the number of lesions and mandatory examinations of patient history and PSA levels. The correlation of PSMA PET to CT and/or MRI morphology, however, is a more assertive analysis tool, since some conditions might present specific anatomical features. Despite the initial thought of PSMA expression in nonprostatic diseases (usually related to endothelial expression in associated neovasculature) as a drawback, these unintentional findings have paved the way for the application of PSMA PET imaging as an additional diagnostic tool for them.

## Data Availability

Yes

## Abbreviations

CT: Computed tomography
FDG: Fluorodeoxyglucose
MRI: Magnetic resonance imaging
PCa: Prostate cancer
PET: Positron-emission tomography
PSMA: Prostate-specific membrane antigen
